# Inflammation and tumor progression: signaling pathways and targeted intervention

**DOI:** 10.1038/s41392-021-00658-5

**Published:** 2021-07-12

**Authors:** Huakan Zhao, Lei Wu, Guifang Yan, Yu Chen, Mingyue Zhou, Yongzhong Wu, Yongsheng Li

**Affiliations:** 1 Department of Medical Oncology, Chongqing University Cancer Hospital, Chongqing, China; 2 Department of Radiotherapy, Chongqing University Cancer Hospital, Chongqing, China

**Keywords:** Tumour immunology, Tumour immunology

## Abstract

Cancer development and its response to therapy are regulated by inflammation, which either promotes or suppresses tumor progression, potentially displaying opposing effects on therapeutic outcomes. Chronic inflammation facilitates tumor progression and treatment resistance, whereas induction of acute inflammatory reactions often stimulates the maturation of dendritic cells (DCs) and antigen presentation, leading to anti-tumor immune responses. In addition, multiple signaling pathways, such as nuclear factor kappa B (NF-kB), Janus kinase/signal transducers and activators of transcription (JAK-STAT), toll-like receptor (TLR) pathways, cGAS/STING, and mitogen-activated protein kinase (MAPK); inflammatory factors, including cytokines (e.g., interleukin (IL), interferon (IFN), and tumor necrosis factor (TNF)-α), chemokines (e.g., C-C motif chemokine ligands (CCLs) and C-X-C motif chemokine ligands (CXCLs)), growth factors (e.g., vascular endothelial growth factor (VEGF), transforming growth factor (TGF)-β), and inflammasome; as well as inflammatory metabolites including prostaglandins, leukotrienes, thromboxane, and specialized proresolving mediators (SPM), have been identified as pivotal regulators of the initiation and resolution of inflammation. Nowadays, local irradiation, recombinant cytokines, neutralizing antibodies, small-molecule inhibitors, DC vaccines, oncolytic viruses, TLR agonists, and SPM have been developed to specifically modulate inflammation in cancer therapy, with some of these factors already undergoing clinical trials. Herein, we discuss the initiation and resolution of inflammation, the crosstalk between tumor development and inflammatory processes. We also highlight potential targets for harnessing inflammation in the treatment of cancer.

## Introduction

Despite the employment in the clinical setting of a series of strategies for cancer treatment (e.g., surgery, chemotherapy, irradiation, and immunotherapy), cancer-related mortality remains one of the leading causes of death worldwide, accounting for 13% of all human deaths.^1^ Because cancer is considered a cell-intrinsic genetic disease, most treatment modalities are focused on killing tumor cells directly, with multidrug resistance of cancer cells being a crucial reason for the low efficacy of cancer therapy.^2,3^ Inflammation has been demonstrated closely associated with all stages of development and malignant progression of most types of cancer, as well as with the efficacy of anti-cancer therapies.^4–6^ In detail, chronic inflammation is involved in immunosuppression, thereby providing a preferred microenvironment for tumorigenesis, development, and metastasis.^7^ Besides, inflammatory responses can be induced by anti-cancer therapies.^8,9^ Acute inflammation contributes to cancer cell death by inducing an anti-tumor immune response, while therapy-elicited chronic inflammation promotes therapeutic resistance and cancer progression.

The correlation between inflammation and cancer was firstly suggested by Rudolf Virchow in the mid-19th century, based on observations that cancer originated in sites of chronic inflammation, and that inflammatory cells were abundant in tumor biopsies.^10^ Nowadays, cancer-related inflammation is considered as a key characteristic of cancer, with a well-established link between chronic inflammation and tumor development.^11^ In fact, chronic, dysregulated, persistent, and unresolved inflammation has been associated with an increased risk of malignancies, as well as the malignant progression of cancer in most types of cancer.^4,5,12^ Moreover, growing evidence have implied that the inflammatory tumor microenvironment (TME) is a key determinant for the therapeutic efficacy of conventional chemotherapy (e.g., radiotherapy and chemotherapy) and immunotherapy.^2,6^ However, acute inflammation induced by exogenous stimulators has been reported to enhance anti-tumor immunity by promoting the maturation and function of dendritic cells (DCs) and the initiation of effector T cells.^13^

Inflammation involving the innate and adaptive immune systems is known to be the protective immune response for maintaining tissue homeostasis by eliminating harmful stimuli, including damaged cells, irritants, pathogens, and sterile lesions.^5,14^ Unlike wound healing and infection, the inflammatory response during cancer development has been demonstrated to be non-resolving.^14^ Furthermore, tumor-extrinsic inflammation is known to be triggered by various factors, including autoimmune diseases, bacterial and viral infections, obesity, smoking, asbestos exposure, and excessive alcohol consumption, all of which have been reported to increase cancer risk and accelerate malignant progression. In contrast, cancer-intrinsic or cancer-elicited inflammation might be caused by cancer-initiating mutations and contribute to tumor progression *via* the recruitment and activation of inflammatory cells.^15–17^ Both extrinsic and intrinsic inflammation are known to result in immunosuppressive TME, thereby providing a preferred condition for tumor development. Once the inflammatory TME is established, inflammatory factors derived from tumor cells or interstitial cells would induce cell proliferation and prolong cell survival by initially activating oncogenes and subsequently inactivating tumor suppressor genes.^15,16^

Owing to the relationship between inflammation and tumor,^13^ harnessing inflammation appears to be an important approach for a more efficient anti-cancer treatment. The powerful chemopreventive effects of non-steroidal anti-inflammatory drugs (NSAIDs), particularly aspirin, have been demonstrated in numerous clinical studies.^18–20^ Administration of statins has also been reported to significantly reduce the risk of development of multiple types of cancer, including breast cancer, colorectal cancer (CRC), and hepatocellular carcinoma (HCC), by exerting anti-inflammatory effects.^21–23^ In addition, increasing the level of specialized proresolving lipid mediators (SPM, e.g., lipoxin A_4_ (LXA_4_) and resolvin D1 (RVD1)) and their synthetic pathways was also shown to significantly inhibit the tumor growth.^24–26^ Moreover, enhancing tumor immunity by blocking inhibitory checkpoints or using chimeric antigen receptor T-cell (CAR-T) immunotherapy has shown promising efficacy in certain cancer types.^27,28^ However, side-effects of these therapies, such as coagulopathy and “cytokine storm” have hindered their full application to cancer therapy,^29,30^ suggesting that reduction of these harmful immunotherapy-generated inflammation events would be beneficial for the outcome of patients with cancer.

In brief, tumor-related chronic inflammation has been shown to promote immunosuppression of the TME and the development of tumor.^13^ Thus, a better understanding of the relationship between the dysregulated inflammation and tumor progression would be conducive toward the development of new strategies for combating tumors, and would enhance the efficacy of immunotherapy, chemo- or radiotherapeutic approaches. In this review, we discuss the initiation and resolution of inflammation, crosstalk between tumor development and inflammatory processes, as well as cancer therapeutic approaches by modulating inflammation.

## The initiation and resolution of inflammation

Inflammation is known to be a protective response of the host against infection and tissue damage, which can prevent the spread of pathogens or promote tissue repair.^31,32^ In the early or acute stages of inflammation, pathogen-associated molecular patterns (PAMPs) are recognized by tissue macrophages or mast cells, activating the secretion of pro-inflammatory cytokines, chemokines, vasoactive amines, and eicosanoids, thereby enhancing the immune response.^33–35^ These pro-inflammatory mediators are known to increase vascular permeability, leading to a massive influx of plasma containing antibodies and other soluble components.^36^ In addition, the injury site has been shown to release a variety of signaling molecules, including chemokines, cytokines, eicosanoids, and adhesion molecules, leading to the recruitment of neutrophils and monocytes.^33,37^ As the inflammatory response progresses, monocytes and lymphocytes accumulate in the inflammation sites to neutralize harmful substances. Subsequently, inflammatory cells undergo apoptosis and cleared by macrophages. In addition, SPM biosynthesis during the resolution of inflammation, have been reported to prevent the infiltration of neutrophils, reduce the secretion of pro-inflammatory mediators, stimulate macrophages to phagocytose apoptotic neutrophils, remove bacteria, and restore tissue homeostasis.^38–41^ At the final stage of the inflammatory cascade, the tissue repair process replaces the inflammatory process, alleviating the inflammatory response and re-establishing tissue homeostasis.^39,40^ Therefore, the inflammatory process involves different types of cells and mediators, which can regulate cell chemotaxis, migration, and proliferation in a highly-programmed manner.

### Acute and chronic inflammation

Inflammation can be divided into two categories according to the length of the disease: acute and chronic inflammation. Acute inflammation is the initial response to harmful stimuli and persists for a couple of days or weeks. The majority of infiltrating inflammatory cells in acute inflammation are granulocytes.^38,42,43^ Chronic inflammation is characterized by the simultaneous occurrence of destruction and healing of tissues. The main infiltrating immune cells in chronic inflammation sites are macrophages and lymphocytes.^44,45^ If the pro-inflammatory stimulus is not eliminated during the acute inflammation process, it will lead to chronic inflammation, autoimmunity, tissue fibrosis, and necrosis. The persistence of inflammatory factors and damage to tissues are the key factors of chronic inflammation.^46,47^ Sustained acute inflammation without obvious symptoms are also known to be a cause of chronic inflammation, such as chronic cholecystitis and chronic pyelonephritis.^48^ Chronic inflammation has also been demonstrated to be induced by chronic intracellular viral infections, such as infection with *Mycobacterium tuberculosis*. These pathogens are less virulent but have been found to cause immune responses with no clinical manifestation of acute inflammation.^49^ Long-term exposure to nondegradable but potentially toxic substances, such as silicosis,^50^ or persistent immune response against self-tissues could cause autoimmune diseases, e.g., rheumatoid arthritis.^41,51^ Moreover, insufficient exercise, obesity, gut microbiota disorders, and an “inflammatory diet” (high in meat and fat, and low in fiber and ratio of omega-3/omega-6 fatty acids) are also known to be incentives of chronic inflammation.^52–54^ Chronic inflammation has been linked to many chronic diseases either directly or indirectly, such as atherosclerosis, myocardial infarction, chronic heart failure, Parkinson’s disease, Alzheimer’s disease, asthma, diabetes, psoriasis, osteoporosis, and cancer.^55–57^ Almost 20% of human cancers and infections have been related to chronic inflammation.^6,58^ Common risk factors associated with cancer development during chronic inflammation are known to include *Helicobacter pylori* infection in gastric cancer, hepatitis B or C infection in HCC, human papilloma virus (HPV) infection in cervical cancer, and so on.^59–61^

### Inflammatory cells

#### Vascular endothelial cells

Vascular endothelial cells are known to play an important role in the inflammatory process. They are widely distributed in the inner side of the vascular cavity, forming a relatively stable barrier, separating the blood from the subcutaneous tissue. In the early stage of inflammation, they have been shown to regulate the permeability of blood vessels and affect the infiltration of inflammatory cells.^62^ During inflammation, leukocyte-synthesized and released TNF-α and IL-1 cytokines have been found to promote the pro-inflammatory phenotype of endothelial cells and fibroblasts through the activation of the TNFR/IL-1 pathway and NF-κB signaling.^63,64^ Activated endothelial cells express adhesion molecules, such as selectins and intercellular adhesion molecule (ICAM)−1, and secrete a large amount of chemokines.^65^ In addition, immobilization of CXC and CC chemokines on endothelial and matrix glycosaminoglycans was reported to create a chemotactic gradient, leading to the recruitment and extravasation of neutrophils and monocytes.^66^ More specifically, CXC chemokines, including CXCL8 (IL-8), macrophage inflammatory protein 2 (MIP-2, known also as CXCL2), complement C5a, leucine, and platelet-activating factor (PAF) have been reported to mediate the process of neutrophil infiltration.^66,67^

#### Neutrophils

Upon an inflammatory stimulus, numerous immune cells are recruited to the site of inflammation. Among these cells, neutrophils constitute the largest circulating leukocyte population in blood and are critical in defending against microbial pathogens infection.^68^ Their rapid recruitment to inflammatory sites is known to occur through a multistep adhesion cascade process.^69^ Initially, circulating neutrophils in circulating blood are “trapped” in blood vessels and migrate along the capillaries to the venule endothelium. This adhesion interaction is known to be mediated by members of the selectin family, such as P- and E-selectins expressed on the surface of endothelial cells. After traumatic stimulation, the surface of vascular endothelial cells rapidly express P-selectin, thus fulfilling the adherence of leukocytes to endothelial cells. The P-selectin glycoprotein ligand 1 (PSGL1) is commonly expressed in all lymphocytes, monocytes, eosinophils, and neutrophils. L-selectin expressed on neutrophils promote their attachment to the surface of the endothelium and sensing of inflammatory mediators, such as CXC chemokines and components of the complement cascade, leading to the activation of integrins.^70^ Subsequently, adhered neutrophils interact with endothelial transmembrane proteins, including platelet endothelial cell adhesion molecule (PECAM)−1, intercellular adhesion molecule (ICAM)−1, vascular endothelial (VE)-cadherin, and members of the junctional adhesion molecule (JAM) family to penetrate the vascular endothelium and migrate to the site of inflammation.^71,72^ Neutrophils display a wide range of roles during the inflammatory process, including phagocytosis of microorganisms, production of reactive oxygen species (ROS), secretion of proteases, and formation of neutrophil extracellular traps (NETs).^73^ These cells are crucial for the resolution of inflammation and reestablishment of tissue homeostasis.^74^ It has been found that wound healing is delayed in neutrophil depletion murine models,^75^ and depletion of neutrophils lead to the exacerbation of autoimmune diseases, such as ulcerative colitis,^76^ suggesting that these cells have pivotal roles during the inflammatory process.

#### Monocytes

The recruitment of monocytes and their differentiation into macrophages are essential for the onset, progression, and resolution of inflammation. During the onset of the inflammation process, the chemokine monocyte chemotactic protein (MCP)1/CCL2 was found to mediate the recruitment of pro-inflammatory monocytes expressing the chemokine receptor CCR2.^77^ As the inflammation progresses, the macrophage colony-stimulating factor (M-CSF), which can promote the differentiation of monocytes to macrophages, was significantly upregulated in the inflammation site.^78,79^ Macrophages have multiple functions and a plastic phenotype in responding to their inflammatory environment: M1 macrophages have a pro-inflammatory phenotype and produce pro-inflammatory factors, whereas M2 macrophages have immunosuppressive effects.^80^ These immunosuppressive macrophages express elevated 15-lipoxygenase (15-LOX) and transforming growth factor (TGF)-β, thus dampening leukocyte trafficking, promoting efferocytosis and wound repair.^81^ In addition, SPM were reported to upregulate microRNAs targeting inflammatory genes in macrophages, thereby downregulating the translation of inflammatory cytokines and chemokines.^82^

#### Mast cells

Mast cells are long-lived tissue-resident immune cells that play a protective role in limiting infections by microorganisms.^83^ They are maintained in constant numbers in healthy tissues, whereas their population increases dramatically during inflammation. Among various receptors, TLRs are the most studied pattern recognition receptors known to interact with a multitude of pathogen-associated molecular patterns from microorganisms or damaged cells. Mast cells have 9 types of TLRs^84^ and express various pro-inflammatory mediators upon activation. For instance, activation of TLR2 has been shown to induce the secretion of TNF, IL-6, IL-13, IL-4, and IL-5, while activation of TLR-4 elicits the expression of TNF, IL-6, IL-13, and IL-1β.^85^ Importantly, mast cells are known to reside in most tissues, especially located in epithelial barriers exposed to external environmental factors, such as the skin, airways, and gut tract. These locations particularly highlight the importance of mast cells in the initiation and propagation of immune responses.^86^ Moreover, activated mast cells have also been reported to release histamine and proteases, promoting the production of pro-inflammatory IL-1 family members, including IL-1, IL-6, and IL-33.^87^

#### T cells

T cells play a crucial role in antiviral responses through the production of cytokines.^88^ T cells are activated during inflammation, and differentiate into various T-cell subsets, including T-helper (Th)1, Th2, Th17, and regulatory T (Treg) cells, depending on the cytokines secreted around the inflammation loci. In particular, Th1 cells are derived following stimulation with interferon (IFN)-γ and TNF-α and secrete IFN-γ, TNF-α, and IL-2, whereas Th2 cells are derived in the presence of IL-4 or IL-10 and secrete IL-4, IL-5, IL-9, and IL-13. In addition, Th17 cells, which secrete IL-17, IL-23, and IL-22, are derived in the presence of TGF-β, IL-1β, and IL-6. In contrast, Treg cells are raised in the presence of TGF-β, and secrete immunosuppressive cytokines, including IL-10 and TGF-β. IL-17 is known to stimulate the production of inflammatory mediators, including TNF-α, IL-6, and IL-1β, whereas Treg cells have been shown to effectively regulate the resolution of inflammation. In addition, CD4^+^ T cells have been reported to promote the production of virus-specific antibodies by activating B cells, whereas CD8^+^ T cells produce IFN-γ and TNF-α and can kill viral-infected cells.^89^ T-helper cells are known to produce a variety of pro-inflammatory cytokines and chemokines by activating NF-κB signaling, recruiting lymphocytes and leukocytes to the site of inflammation, where all these immune cells express and secrete additional chemokines and cytokines amplifying the inflammatory process in response to viral infections.^90^

#### Dendritic cells

DCs are antigen-presenting cells that sense microbial and capture, process, and present antigens to lymphocytes.^91^ They stimulate the activation and proliferation of antigen-specific T and B lymphocytes to initiate the adaptive immune response.^92,93^ DC activation leads to the secretion of pro-inflammatory mediators which include antimicrobial mediators and chemokines, and recruit more immune cells to the site of infection. Also, DCs regulate T cells differentiation into distinct subsets such as Th1, Th2, Th17, and Treg cells.^94,95^

#### Myeloid-derived suppressor cells

Myeloid-derived suppressor cells (MDSCs) are immature myeloid cells involved in the regulation of acute and chronic inflammatory conditions such as autoimmune and infectious diseases.^96^ It is known that MDSCs can be recruited into inflamed tissues where they trigger the resolution of inflammation.^96^ Various studies show MDSCs suppress the activity of immune cells through different mechanisms involving the degradation of L-arginine, the production of ROS, and the secretion of anti-inflammatory cytokines like IL-10 and TGF-β.^97^ In addition, MDSCs can inhibit T cell activity by downregulating the pro-inflammatory cytokines, such as IL-12 and prostaglandin E_2_ (PGE_2_).^98^

#### Basophils and eosinophils

Although represent only about 0.5% of all leukocytes in human blood, basophils are important immune cells of both innate and acquired immunity.^99,100^ Basophils release a variety of pro-inflammatory mediators and cytokines such as IL-4, IL-13, IL-6, IL-9, CCL5, granulocyte-macrophage colony-stimulating factor (GM-CSF), MIP-1, and monocyte chemoattractant protein-1 (MCP-1/CCL2).^101^ It was demonstrated that basophils can be activated by IL-18 and IL-33.^102^ Upon stimulation they undergo degranulate, release and synthesize pro-inflammatory, vasodilative, chemotactic, and cytotoxic substances. These cells are crucial for allergy and inflammation. Eosinophils are other innate immune leukocytes and play important roles in host defense against parasitic, viral, fungal, and bacterial infections.^103^ Moreover, there is emerging evidence that eosinophils have an immune regulatory and homeostatic function. Eosinophils constitutively express 12/15-LOX which is a key enzyme for the synthesis of SPM, thereby promoting the resolution of inflammation.^104^

#### Natural killer and B cells

Natural killer (NK) and B cells are also involved in the inflammatory process. For instance, NK cells are important immunosurveillance cells that detect infected, transformed, or stressed cells with their activating receptors NKG2D and NKp46.^104^ Once activated, NK cells become cytotoxic and release lytic granules (perforin, granzymes) or induce death signals (e.g. TNF-related apoptosis-inducing ligand (TRAIL)/TRAL-R, Fas ligand (Fas-L)/Fas), thereby kill microorganisms.^105^ B cells are transformed into plasma cells and secrete antibodies to kill microorganisms, in a mechanism called antibody-dependent cell-mediated cytotoxicity (ADCC). Macrophages, B cells, and DCs are also known to activate T cells through antigen cross-presentation.^106,107^ However, the chemotactic mechanisms driving the recruitment of monocytes to repair tissues as the inflammation progresses, are not well understood.^106^ Still, the phenotype of monocytes at the site of inflammation has been demonstrated to be dynamically regulated by inflammatory cytokines and mediators. These pro- and anti-inflammatory factors were reported to lead to the production of subpopulations of macrophages with different functional characteristics that regulated the activity of fibroblast cells, matrix metabolism, angiogenesis, and promoted tissue repair processes.^78,79^

### Pro- and anti-inflammatory factors

During the inflammatory response, an extremely complex regulatory network takes place, involving pro-inflammatory cytokines, pro-inflammatory cytokine-releasing cells, and pro-inflammatory cytokine target cells.^108^ In addition to pro-inflammatory cytokines, there exist many other inflammatory mediators, which are small molecule compounds closely related to the vascular response, nervous system response, and cell hyperplasia response.^33^ Various inflammatory factors are produced by specialized immune cells, especially tissue-resident macrophages and mast cells, or cells present in local tissues.^33^ Some inflammatory mediators (e.g., histamine and serotonin) are known to be expressed and stored in the granules of mast cells, basophils, and platelets.^35^ Whereas, other mediators are formed and circulate in the plasma as inactive precursors. The plasma concentration of these mediators has been demonstrated to increase significantly during acute inflammation due to the increased secretion of precursors.^33,109^ Inflammatory mediators can be divided into seven groups based on their biochemical properties: vasoactive amines, vasoactive peptides, cytokines, chemokines, fragments of complement components, lipid mediators, and proteolytic enzymes.

Vasoactive amines, including histamine and 5-hydroxytryptamine (5-HT), are mainly released by mast cells. Histamine synthesis occurs through the decarboxylation of the amino acid histidine by an enzyme called L-histidine decarboxylase (HDC), which has been found in mast cells, basophils, and gastric mucosal cells. Likewise, 5-HT is produced by the decarboxylation of tryptophan, and is stored in the granules of mast cells.^110^ Mast cells have been shown to release histamine and 5-HT when stimulated by physical factors, such as trauma, heat, immune response, and complements. These mediators have complex effects on the vascular system, including increased vascular permeability, vasodilation or vasoconstriction.

Vasoactive peptides, such as substance P, can be stored in secreted vesicles either in their active form or as inactive precursors (e.g., kinin, fibrinopeptide A/B, and fibrin degradation products) that can be processed by proteolytic enzymes.^110^ Substance P is released by sensory neurons, and has been reported to cause the degranulation of mast cells.^111^ Other vasoactive peptides are known to be produced by proteolysis of hageman factors, thrombin, or plasmin, and they have been found to cause vasodilation and increase vascular permeability directly or indirectly by inducing the release of histamine from mast cells.^112^ Hageman factors play a key role in these reactions, acting both as sensors of vascular damage and as inducers of inflammation.^113^

Cytokines are the major signaling molecules released by inflammatory cells and involved in multiple functions. They are classified into pro-inflammatory cytokines (IL-1, IL-6, IL-15, IL-17, IL-23, TNF-α, and IFN-γ) and anti-inflammatory cytokines (IL-4, IL-10, IL-13, and TGF-β).^114^ Among them, TNF-α, which is mainly produced by macrophages and mast cells, is one of the earliest and most important inflammatory mediators. TNF-α is known to have multiple roles in the inflammatory response, including the activation of inflammatory cytokines coded by the NF-κB signal pathway, adhesion molecules, gene expression of prostaglandin synthesis pathway enzymes (e.g., cyclooxygenase-2 (COX2)), induction of nitric oxide synthase (iNOS), leading to the activation of endothelium and white blood cells.^15,115–118^ It has also been reported to activate neutrophils and lymphocytes, increase the permeability of vascular endothelial cells, regulate the metabolic activities of other tissues, and promote the synthesis and release of other cytokines.^119,120^ Accordingly, IL-1 and IL-6 are well-known interleukins that participate in the production of ROS and reactive nitrogen species (RNS), and in the synthesis of inflammatory molecules, such as chemokines, integrins, and matrix metalloproteinase (MMP). Macrophages and T cells are the major cell sources of these interleukins. Both IL-1 and IL-6 bind to their respective IL-1R and IL-6R receptors, leading to the activation of NF-κB and JAKs-STAT pathways. Besides, IL-6 is also known to induce the differentiation of B cells for production of antibodies, and promotes the activation, proliferation, and differentiation of T cells.^121,122^

Chemokines are a family of small (generally 8–10 kDa) signaling peptides that have an important role in the recruitment of inflammatory cells during inflammation.^123^ They are divided into four families (C, CC, CXC, and CX_3_C) based on the spacing of their N-terminal cysteines.^124^ The major secreted chemokines during inflammation, which direct leukocyte migration and influence the activity of infiltrating immune cells, belong to the CC and CXC families. Chemokines have been shown to bind to their G protein-coupled cell-surface receptors (GPCRs) to exert their cellular effects, such as cell movement and activation.^125–127^ Chemokines are mainly released by innate immune cells including neutrophils, mast cells, and eosinophils.^127,128^ For instance, CCL2 is a chemokine important for the recruitment of monocytes in response to stimuli. Monocytes have been found to migrate to the inflammatory site following CCL2 gradients orchestrated by vascular endothelial cells in response to PAMPs.^129,130^ Likewise, CXCL12, also known as stromal cell-derived factor-1 (SDF-1), is another well-studied chemokine that contributes to tissue repair by mobilizing mesenchymal stem cells (MSCs) to injury sites trough binding to CXCR4.^131^ More specifically, binding of CXCL12 to CXCR4 has been reported to lead to the activation of G protein-coupled receptor (GPCR) downstream signaling, such as phosphatidylinositol-3-kinase (PI3K)/mechanistic target of rapamycin (mTOR) and mitogen-activated protein kinase kinase (MEK)/extracellular signal-regulated kinase (ERK) signaling, thereby promoting tissue repair.^132^

The C3a, C4a, and C5a complement fragments are small soluble peptide fragments that play key roles in the regulation of inflammation.^133^ They are produced through several complement activation pathways and are known to promote the recruitment of granulocytes and monocytes, as well as induce the degranulation of mast cells.^134,135^ The activation of the complement system is triggered by either the classical, the lectin, or the alternative pathway.^136^ These pathways share the same proteolytic cascade processes, thereby promoting the cleavage of inactivated complement components into active peptide fragments.^137^ In this regard, the C3 and C5 complement components are cleaved into the C3a, C3b, C5a, and C5b fragments.^138^ In particular, C3a is known to be an anaphylatoxin with chemotactic activity and participates in the production of pro-inflammatory cytokines. Meanwhile, C3b produced by C3 convertases has been shown to function as a constituent of C5 convertases. The C5b fragment bind to target cells, allowing the assembly of the membrane attack complex (C5b-9 or MAC), thus leading to the lysis of target cells. In addition, the C3a and C5a complement fragments have been demonstrated to orchestrate inflammatory responses by binding to their C3aR and C5aR receptors, respectively.^139,140^ Once these complement fragments bind to their receptors, target cells show various responses, such as migration, antigen presentation, and the production of inflammatory mediators.^141,142^

Lipid mediators constitute one of the most important category of mediators of inflammation.^143^ Following the activation of cells by Ca^2+^ ions, cytoplasmic phospholipase A2 generates arachidonic (AA) and lysophosphatidic acid from phosphatidylcholine. Subsequently, AA is metabolized either by cyclooxygenases (COX1 and COX2) to produce prostaglandins and thromboxanes, or by lipoxygenases (LOXs) to produce leukotrienes and lipoxins.^144–146^ The PGE_2_ and prostacyclin I_2_ (PGI_2_) in turn cause vasodilatation, with PGE_2_ being also an effective stimulator of hyperalgesia and fever.^144^ In addition, AA-derived lipoxins and dietary omega-3 fatty acid-derived resolvins and protectins have been reported to inhibit inflammation and promote the resolution of inflammation, and tissue repair.^146,147^

Several proteolytic enzymes, including elastin and cathepsin, have multiple roles in inflammation, partly by degrading extracellular matrix (ECM) and basement membrane proteins.^148–150^ These proteases play an important role in many processes, including host defense, tissue remodeling, and leukocyte migration. Elastin is a dominant ECM protein in the lung, and plays a significant role in cardiovascular inflammation and calcification.^151^ Fragments of elastin have been shown to induce the differentiation of Th1 cells and enhance the release of IFN-γ from T cells.^152^ Cathepsins are lysosomal proteases composed of 11 members, including cathepsin B, C, F, H, K, L, O, S, V, W, and Z in humans,^153,154^ and have been reported to be involved in immune modulation through the proteolysis of the ECM and extracellular or membrane-bound proteins.^155^ For instance, cathepsin S is a cysteine protease involved in the cleavage of elastin and generation of bioactive elastin peptides.^156^ Cathepsin B (CatB) is known to have a role in the production of mature IL-1β and TNF-α,^157^ while cathepsin K (CatK) contributes to the activation of TLR9.^158,159^

The regulation of inflammatory mediators can occur at multiple levels, including transcription, mRNA translation, posttranslational modification, and mRNA degradation.^160^ The posttranscriptional regulation has been shown to play an important role in controlling the expression of these mediators for the normal and efficient initiation and resolution of inflammation. The mRNA of many inflammatory mediators has been shown to be unstable, partly because of the presence of AU-rich elements in their 3′-untranslated regions. Moreover, it has been found that binding of many RNA binding proteins to these AU-rich elements could lead to the regulation of the stability or translation of mRNA. For example, the infusion of *E. coli* in primates has been demonstrated to trigger the rapid release of TNF and other inflammatory cytokines, with their serum levels reaching a peak in 90 min and then quickly disappearing.^161,162^ The mRNA transcripts encoding these proteins contain regulatory elements that direct their degradation or translational inhibition. When these transcripts are induced, they synthesize proteins in a short time interval, and then are destroyed or silenced, thus preventing the overexpression of pro-inflammatory proteins.^163–165^

IFN-γ is another important pro-inflammatory factor in the inflammatory process that has been reported to both activate and inhibit the mRNA transcription of inflammatory genes.^166^ For example, in macrophages, IFN-γ was shown to induce the transcription of the pro-inflammatory gene ceruloplasmin,^167^ and also initiated the formation of IFN-γ-activated translation inhibitor (GAIT) complexes, which interact with the GAIT element located on the 3′-UTR of mRNAs encoding a variety of pro-inflammatory genes, including ceruloplasmin.^168^ Binding of the GAIT complex to the GAIT element in the mRNA inhibits the protein translation machinery and consecutively protein synthesis. As a result, ceruloplasmin has been found to be secreted by IFN-γ-activated macrophages for about 16 h, after which its levels are decreased.^169^ This fine adjustment of inflammatory mediators ensures the timely termination of the inflammatory process and returns the expression levels of pro-inflammatory factors and proteins to baseline levels.

### Inflammation resolution

In order to prevent the progression from acute-resolving to persistent-chronic inflammation and allow organs to restore homeostasis, the inflammatory reaction must be actively resolved, to prevent further tissue damage.^170,171^ Historically, it was believed that the resolution of inflammation was a passive process involving the dilution of chemokine gradients over time, thus stopping the recruitment of circulating leukocytes to the site of injury.^172^ However, extensive work over the past few decades has revealed that the resolution of inflammation is a programmed active process, and deficiency in any of its components might lead to overactive, uncontrolled chronic inflammation. With the advancement of lipidomics and metabolomics, Serhan et al. showed that the resolution phase of inflammation is regulated by a class of enzymatically produced SPM.^36,173^ They also introduced the quantitative resolution indices (defined as follows: *T*_max_: time point when PMN infiltration to maximum; *Ѱ*_max_: PMN maximum number; *T*_50_: time point when PMNs reduction to half of *Ѱ*_max_; *Ѱ*_50_: 50% of *Ѱ*_max_; *R*_i_: resolution interval, time interval from *T*_max_ to *T*_50_; *K*_50_: the rate of PMN reduction from *T*_max_ to *T*_50_), which indicated reduced PMN infiltration and shortened resolution interval after SPM biosynthesized.^174^

**Fig. 1:**
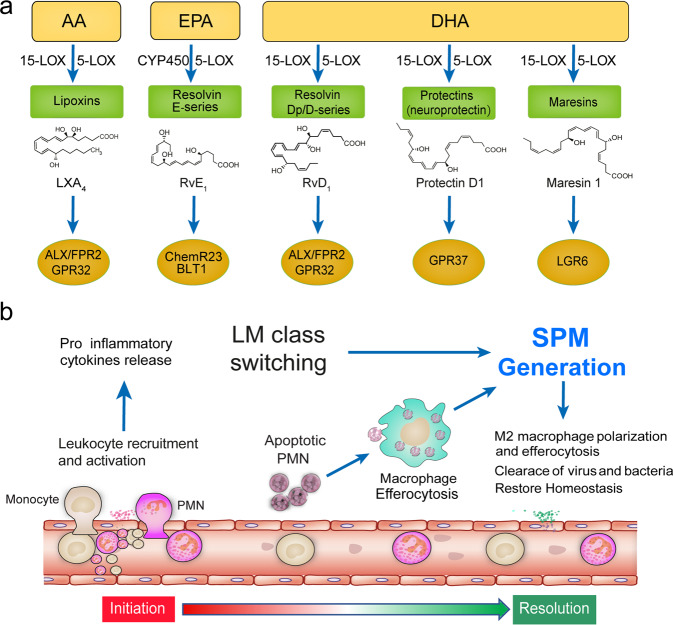
SPM biosynthesis and their roles in the resolution of inflammation. **a** SPM including lipoxins, E-series resolvins, D-series resolvins, protectins (neuroprotectin D1), and maresins are biosynthesized from arachidonic acid (AA), eicosapentaenoic acid (EPA), and docosahexaenoic acid (DHA). The main structures of these SPM and their receptors are depicted. **b** Anti-inflammatory lipid mediators (LM) class are produced to help restore tissue homeostasis during the resolution of inflammation

Upon inflammation initiation, the pro-inflammatory lipid mediators (LM) are produced, whereas during the resolution of inflammation, the SPM are abundantly biosynthesized, i.e., LM class switching occurs (Fig. 1). SPM have been shown to not only function as signals for the termination of the inflammatory response, but also promote macrophages to engulf dead cells to accelerate the resolution of inflammation. Removal of apoptotic neutrophils by macrophages is a prerequisite for macrophage efferocytosis, which has been reported to coincide with the biosynthesis of SPM, reducing the expression of pro-inflammatory lipid mediators and cytokines.

Lipoxins are a class of metabolite derivatives of AA *via* the lipoxygenase pathway. In the vascular cavity, leukocyte-derived 5-LOX is known to catalyzes the synthesis of leukotriene A4 (LTA_4_) which is then catalyzed by platelet-derived 12-LOX to produce LXA_4_ or LXB_4_.^175^ Lipoxins have also been found to be catalyzed by 15-LOX in epithelial cells, monocytes, and eosinophils to produce intermediate products, followed by their catalysis by 5-LOX in neutrophils to produce LXA_4_ or LXB_4_. Serhan et al. discovered that aspirin-mediated acetylation of COX2 inhibited the production of prostaglandin but led to the conversion of AA to 15(R)-hydroxyeicosatetraenoic acid (15(R)-HETE), a substrate used for the synthesis of 15-epi-lipoxins (AT-lipoxins). In addition, lipoxins have been reported to promote the resolution of inflammation through activating lipoxin receptor (ALX)/N-formyl peptide receptor (FPR)−2 receptors to antagonize pro-inflammatory mediators, resulting in decreased recruitment of leukocytes and deactivation of NF-κB, decreased production of superoxide, and diminished production of pro-inflammatory chemokines/cytokines.^176,177^

Resolvins are another series of important endogenous SPM. Depending on their source, they either contain E-series (RvE) derived from eicosapentaenoic acid (EPA), D-series (RvD) from docosahexaenoic acid (DHA) and aspirin-triggered resolvin D (AT-RvD1-RvD6), or Dp series (RvD_n-3DPA_) derived from docosapentaenoic acid (DPA).^178–180^ Resolvins are synthesized through interactions between the activities of aspirin-acetylated COX2 and LOX in endothelial cells and leukocytes. In particular, RvE1 is known to activate downstream pathways by binding to ERV1/ChemR23, leading to the inhibition of the NF-κB pathway in inflammatory cells.^181^ Meanwhile, RvD1 and RvD3 exert their bioactions through binding to ALX/FPR2 and DRV1/GPR32, respectively, whereas RvD2 and RvD5 activate their DRV2/GPR18 and DRV1/GPR32 receptors, respectively.^178,182,183^ Of interest, the activation of the RvE1-ERV1/ChemR23 axis has been shown to promote the apoptosis and macrophage-mediated phagocytosis of neutrophils, while reducing the production of pro-inflammatory cytokines.^184–186^ Recently, we also found that RvD_P_5 inhibited the infiltration of neutrophils and promoted the phagocytic function of macrophages through the ALX/FPR2 receptor.^187^

In addition to lipoxins and resolvins, additional families of SPM, namely protectins and maresins have also been identified. Protectin is derived from metabolites of DHA epoxidation. Because of its potent protective effect in central neurons, protectin D1 is also called neuroprotectin.^174,188,189^ The synthesis of protectins and maresins is known to be catalyzed by 15-LOX and 5-LOX.^190^ Protectin D1 has been shown to promote macrophage phagocytosis of apoptotic polymorphonuclear leukocytes (PMNs) and regulate the infiltration of leukocytes.^191^ In macrophages, DHA has been demonstrated to generate intermediate 14S-HDHA from the lipoxygenation of 12-LOX, subsequently generating maresins, including MaR1 and MaR2, through epoxidation or hydrolysis.^192,193^ Moreover, it has been shown that cytochrome oxidase could also convert DHA into 19,20-EDP, which could also be quickly converted by soluble epoxide hydrolase into the inactive 19,20-Di DHPA metabolite. Maresin 1 is the first member of the maresin family and has been found to restrict the infiltration of neutrophils, enhance the phagocytosis of apoptotic neutrophils and necrotic cells by macrophages, downregulate the production of pro-inflammatory mediators, inhibit the activation of NF-κB, and increase the content of regulatory T cells. Maresin 1 has also been demonstrated to increase the level of intracellular cyclic adenosine monophosphate to promote the resolution of inflammation; however, the receptors that interact with maresins and their mechanisms of action remain unclear.^194,195^

Recently, a group of peptide-conjugated SPM, such as protectin conjugates in tissue regeneration (PCTRs), maresin conjugates in tissue regeneration (MCTRs), and resolvin conjugates in tissue regeneration (RCTRs) have been discovered.^196–198^ These SPM are biosynthesized from DHA. PCTR1, an endogenous novel peptide-conjugated SPM that exerts anti-inflammatory and proresolving functions during infection have been recently discovered.^199,200^ More specifically, PCTR1 is produced from DHA in leukocytes and reduce pro-inflammatory factors in serum and improve the survival rate of mice during LPS-induced acute inflammation. In addition, PCTR1 reduced the levels of LPS-induced serum linoleic acid (LA), AA, and PGE_2_ via the activation of ALX.^199^ Moreover, PCTR1 promotes the conversion of LA to AA through the upregulation of LPS-inhibited fatty acid desaturase 1/2 (FADS1/2) and elongation of very long-chain fatty acids 2 (ELOVL2), and the inhibition of the expression of phospholipase A2 (PLA2) resulted in the increased intrahepatic content of AA. Similar to PCTRs, MCTRs act as anti-inflammatory and proresolving agents and contribute to host defense, organ protection, and pain modulation.^201,202^ MCTRs are produced by macrophages and participate in phagocytosis and tissue repair and regeneration.^203^ However, the signaling mechanisms underlying MCTRs functions have not yet been fully established. RCTRs are new chemical signals that play a role in inflammation resolution and tissue regeneration.^204^ RCTRs stimulate macrophage phagocytosis and efferocytosis of apoptotic PMNs, limiting PMN chemotaxis and infiltration, and exert anti-inflammatory and proresolving actions during resolution of inflammation.

The important protective actions of SPM in both acute inflammation (e.g., sepsis,^199^ lung injury,^180^ and ischemia-reperfusion injury^200^) and chronic inflammation (e.g., asthma^205^ and Alzheimer’s disease^206^) have been widely reported. These SPM are known to display protective effects through direct antimicrobial actions in host defense or indirectly by controlling the pathogen-mediated inflammation. For instance, the production of endogenous protectin D1 was shown to be increased during the infection of hosts with influenza viruses to directly inhibit the pathogenicity of influenza by interacting with the RNA replication machinery. Accordingly, insufficient upregulation of protectin D1 led to more efficient viral replication and host demise, whereas treatment of the host with exogenous protectin D1 could restore the inhibition of viral replication and improve host survival. Protectin D1 has pivotal roles in regulating the resolution of inflammation through limiting further recruitment of neutrophils, promoting macrophage clearance of apoptotic neutrophils and efferocytosis, accelerating tissue regeneration, and reducing pain and viral pathogenicity.

The coronavirus disease (COVID-19), an infection caused by a novel ssRNA betacoronavirus (SARS-CoV-2), has spread worldwide and already affected the population in more than 180 countries. Almost all patients with COVID-19 are clinically presented with fever, cough, and dyspnea.^207,208^ Moreover, infection with SARS-CoV-2 has been associated with systemic inflammation, and increased serum levels of inflammatory cytokines and chemokines, including IL-1, IL-7, IL-8, IL-9, IL-10, GM-CSF, and IFN-γ, which have been associated with disease severity and death.^209–211^

As mentioned above, SPM play an important antimicrobial protective role during infection and control pathogen-mediated inflammation, with deregulation of protectin D1 leading to viral replication and systemic inflammation. Thus, we speculated that SARS-CoV-2 might be able to suppress the production of SPM to facilitate its replication, and treatment with exogenous SPM, such as protectin D1 might inhibit its replication, prevent the subsequent cytokine storm, and improve survival rate.^212^ However, whether the production of SPM is changed during infection with SARS-CoV-2 remains unknown. In addition, whether the mechanism by which SARS-CoV-2 might suppress the production of endogenous SPM is to be evaluated. Further investigations of the functional role of SPM in patients with COVID-19 are imperative before SPM could be applied as potential agents.

### Inflammation and immunity

The TLR, NOD-like receptor (NLR), and retinoic acid-inducible gene-like receptor (RLR) families are 3 major pathogen sensor families of innate immunity.^213,214^ The binding of pathogenic or endogenous dangerous factors to these receptors, including TLR and NLR is known to activate a variety of downstream intracellular signaling pathways, leading to the release of a plethora of pro-inflammatory mediators, including cytokines, chemokines, leukotrienes, and eicosanoids. Members of the TLR family can identify bacteria, viruses, fungi, and protozoa. The function of NLR is to detect bacteria, whereas the function of RLR is to sense viruses. These innate immune receptors are essential for the protection of the host from bacterial, viral, fungal, and protozoan infections, as well as in response to cellular stress. Despite the diversity of the TLR family, all members are known to be involved in the inflammatory response and the progression of certain inflammatory diseases, such as atherosclerosis.^214^ Eleven TLRs (TLR1~TLR11) have been identified in human cells, of which TLR1, TLR2, TLR4, TLR5, TLR6, TLR10, and TLR11 are expressed on the cell surface, whereas TLR3, TLR7, and TLR9 are expressed in the cytoplasm.^214^ Briefly, TLRs have 3 structural features: (1) an extracellular region composed of leucine; (2) a transmembrane region; (3) and a cytoplasmic region homologous to the IL-1 receptor, namely the Toll/interleukin-1 receptor (TIR), which is essential for the activation of its downstream signaling pathway.^215,216^ The first step after the activation of TLRs is their dimerization or synergy with other receptors, as well as their redistribution and aggregation on the cell surface. The downstream signaling pathways of TLRs include myeloid differentiation factor (MyD88), IL-1R-related protein kinase (IRAK), TRAF6, TAK1, TAB1, and TAB2. Studies have shown that there are 2 signaling pathways involved in the process of the transduction of the TLR signal, namely the MyD88-dependent and MyD88-independent pathways.^217,218^ Activation of TLR has been found to promote the effects of IRAK (IL-1RI-related protein kinase) 4 and IRAK1 through the recruitment of MyD88 adaptor molecules.^219^ More specifically, IRAK4 was reported to phosphorylate IRAK1, with IRAK1 further interacting with TRAF6 to form a complex, leading to the phosphorylation of TAK1 and TAB2. Then, TAK1 was shown to phosphorylate the inhibitory kappa B kinase (IKK) complex, leading to the activation of the NF-κB transcription factor and promoting the production of inflammatory cytokines, adhesion molecules, and prostaglandins.^220^ Both TLR3 and TLR4 were reported to interact with 2 TIR adaptor proteins, TIRAP and TRIF, independent of the MyD88 adaptor protein.^221^ Although TIRAP plays a role in the signaling pathways of TLR2 and TLR4, it does not participate in the signaling pathways of other TLRs. In contrast, TLR3 and TLR4 could be directly linked to TRIF, inducing the transduction of downstream factors without passing through MyD88.^222^

The NOD-like receptors are pattern recognition receptors in the cytoplasm. The structural features of NLRs are as follows: (1) the central nucleotide-binding oligomerization region (NACHT), which is very important for the oligomerization and activation of NLRs, is a structure shared by the NLR family; (2) the N-terminal effector binding region, that is, the N-terminal protein-protein interaction domain, such as caspase activation and recruitment domain (CARD); and (3) the C-terminal enrichment leucine-containing repeats (LRRs).^223,224^ The NLR family consists of 22 types of intracellular pattern recognition molecules, which are distributed in a variety of tissue cells, including monocytes, macrophages, T cells, B cells, dendritic-like cells of the small intestine, and Paneth cells. Human NLRs are divided into the following 5 categories: NLRA, NLRB, NLRC, NLRP, and NLRX.^225^ It has been shown that NOD1 and NOD2 recruit receptor-interacting protein (RIP)-2 through CARD-CARD interactions, thereby activating the NF-κB and mitogen-activated protein kinase (MAPK) signaling pathways. The combination of the PYD-containing NLRP protein and CARD-containing apoptosis-associated speck-like (ASC) protein has been shown to cause the activation of caspase-1, promoting an inflammatory reaction.^226^ In addition, large amounts of NLR could form inflammasomes. An inflammasome is a multiprotein complex, including NLRs, the ASC intracellular adaptor protein, and caspase-1, which is known to regulate the processing and activation of IL-1β, IL-18, IL-33, and other pro-inflammatory cytokines, and participates in the activation of the innate immune system.^227^ As a result, a complex network is formed between NLR members and inflammatory factors to synergistically regulate the immune response and strengthen the inflammatory response and antimicrobial ability. Excessive activation of NLRP3 or gene mutations have been reported to cause severe inflammatory diseases, such as familial cold-induced autoinflammatory syndrome (FCAS), Muckle-Wells syndrome (MWS), and neonatal onset multisystem inflammatory disorder or chronic infantile neurologic cutaneous and articular syndrome (NOMID/CINCA).^228^

In addition to promoting the maturation and extracellular release of the IL-1β and IL-18 pro-inflammatory cytokines, the activation of the inflammasome could also induce pyroptosis.^229^ Pyrolysis, also known as cell inflammatory necrosis, is a kind of programmed cell necrosis, which is manifested by the continuous expansion of cells until the rupture of the cell membrane, which causes the release of cell contents and activates a strong inflammatory response.^230,231^ The cysteine protease caspase-1 is known to cut the linker between the amino and carboxyl ends of gasermin D (GSDMD), thereby regulating cell pyrolysis.^232^ Recent studies have shown that GSDMD is associated with familial mediterranean fever (FMF),^233^ neonatal multiple inflammatory disease,^234^ nonalcoholic steatohepatitis^235^, and multiple sclerosis in murine models.^236^ In addition, the NLR protein inflammasome-mediated inflammatory response has also been involved in the occurrence and development of certain tumors.^237^ For example, NLRP3 was involved in the inflammatory response caused by anti-tumor drugs.^238,239^ Therefore, these proteins could become targets for the future development of novel drugs and improved treatment approaches.

It is widely known that NF-κB is a family of key transcription factors participating in innate immunity and inflammation, and also involved in the occurrence and development of tumors.^240^ There are 5 proteins in this family in mammals, namely: RelA p65), RelB, c-Rel, NF-κB1 (p50), and NF-κB2 (p52). Their N-terminus has a highly conserved Rel homology region (RHR).^115^ The most common NF-κB dimer is the heterodimer composed of RelA and p50. The NF-κB signaling pathway is activated by extracellular signaling factors, including TNF-α, IL-1β, IL-2, IL-6, IL-8, IL-12, iNOS, COX2, chemokines, adhesion molecules, colony-stimulating factor, and many more.^241,242^ The activation of NF-κB results in the phosphorylation and degradation of inhibitors of NF-κB (IκBs), and the subsequent nuclear translocation of NF-κB and upregulation of numerous pro-inflammatory chemokines and cytokines, such as the IL-1, IL-6, IL-8, and PGE_2_, further promoting the inflammatory response.^243–246^ In addition, studies have shown that one of the important functions of NF-κB in tumor cells is to promote cell survival through the induction of the expression of antiapoptotic genes, such as BCL2, and promotion of the expression of the hypoxia-inducible factor-1α (HIF-1α).^126,246^ These cumulative evidence have linked innate immunity to inflammation and hypoxia. Studying the role of NF-κB in leukocytes infiltrated in the site of inflammation will further strengthen our understanding of the link between immunity and inflammation.

The activation of the NF-κB pathway has been demonstrated to be rapidly induced by viral and bacterial infections, necrotic cell components, and pro-inflammatory cytokines during immune responses.^247^ However, the NF-κB pathway is also known to accelerate cell proliferation, inhibit apoptosis, and promote cell migration and invasion. Notably though, in the TME, NF-κB is constitutively activated, promoting the expression of cytokines, chemokines, and growth factors.^248^ These results highlighted the important roles of NF-κB in inflammation and cancer progression.

## Inflammation roles in cancer: promoting VERSUS inhibiting

As mentioned above, inflammation has been demonstrated to not only promote the immune response but also lead to immune surveillance. The innate and adaptive immunity involved in the inflammatory response were also shown to play an important role in cancer initiation, progression, and metastasis.^5^

The acute inflammatory response is the first line of defense against external infection or injury, promoting innate and adaptive immune responses. The innate immune system consists of evolutionary diversified hematopoietic cells, such as neutrophils, macrophages, DCs, mast cells, and so on.^249^ These cell populations are known to participate in the phagocytosis of pathogens, microorganisms, and necrotic substances, thereby mediating the resolution of inflammation. Moreover, as antigen-presenting cells, DCs and macrophages have also been shown to provide specific antigens to T cells for recognition and activation of the adaptive immune response.^250^ Therefore, acute inflammation could eliminate pathogens and protect the body from infections.

However, if the acute inflammatory reaction does not resolve in time, it could be transformed into chronic inflammation resulting in an immunosuppressive microenvironment with a large number of immunosuppressive cells (M2 macrophages, MDSCs, Treg cells, etc.) and cytokines.^5,15^ These changes have been reported to promote the activation of oncogenes, DNA and protein damage, release of ROS, and affect multiple signaling pathways including NF-κB, K-RAS, and P53, leading to chronic diseases including cancer.^5^ In addition, epigenetic alterations, such as DNA methylation, histone modification, chromatin remodeling, and noncoding RNA, play an important role in the transformation of inflammation into cancer as well as in the occurrence, development, invasion, metastasis, and drug resistance of cancer.^247,251–254^ Particularly worth mentioning is the histone lactylation in macrophages that might promote inflammatory resolution and tumor immune escape,^251,255–258^ but whether lactylation could modify other proteins and their effects on protein functions remain unknown. Moreover, lactic acids in the inflammatory microenvironment are known to play an important role in promoting the progression of inflammation and cancer *via* acting on immune cells (such as cytotoxic T cells (CTLs), DCs, and APCs),^259–261^ and immunosuppressive cells (such as M2-macrophages, MDSCs, and Treg cells).^262–264^

**Fig. 2:**
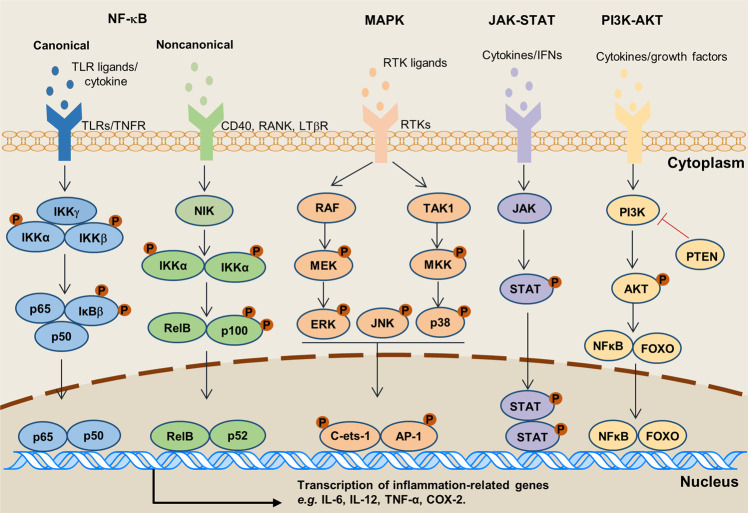
Inflammatory signaling pathways involved in cancer development. Intracellular signaling pathways involved in inflammation and tumor development are activated *via* distinct receptors at the cell membrane. Subsequent downstream signaling events activate several well-characterized pathways: NF-κB, MAPK, JAK-STAT, and PI3K-AKT. These pathways regulate various inflammatory factors

Meanwhile, gene mutations would lead to abnormal cellular proliferation, but immune cells could recognize specific antigens on these tumor cells, and stimulate immune response to clear them. Multiple inflammatory factors and signaling pathways, such as 5-LOX, COX-2, TGF-β, and VEGF are well-known molecules linking inflammation and chronic diseases.^252^ What’s more, the dysregulation of inflammatory molecules or factors is often caused by aberrant inflammatory pathways that including NF-κB, MAPK, JAK-STAT, and PI3K/AKT, etc (Fig. 2). For instance, more than 500 cancer-related genes are regulated by the NF-κB signaling pathway.^247^

**Fig. 3:**
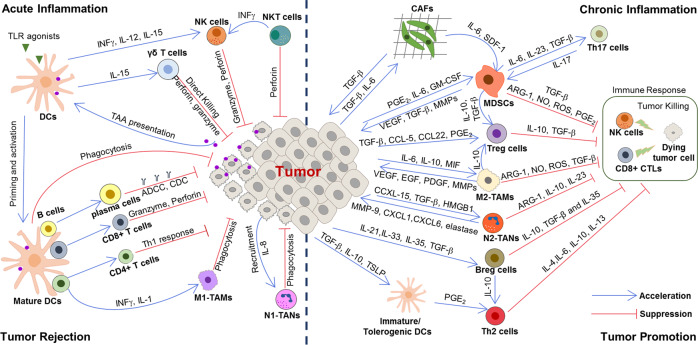
The relationship between inflammation and cancer development. During acute inflammatory responses (left panel): after tumor antigen uptake or activation by TLR agonist, mature DCs can regulate anti-tumor immune responses by inducing inflammatory responses *via* multiple mechanisms, such as cross-presenting the tumor antigens and priming tumor-specific CD8^+^ T cells, polarizing immune cells toward tumor suppression (e.g., M1 polarization of TAMs), recruiting NK cells which can sustain T-cell responses. However, if the acute inflammatory reaction does not resolve in time, it subsequently transforms into chronic inflammation (right panel). In this microenvironment, cancer cells can not only hijack DCs to prevent TAA presentation, but also recruit a large number of immunosuppressive cells (e.g., MDSCs, Treg cells, Breg cells, M2-TAMs, N2-TANs, and Th2 cells) by secreting various cytokines, chemokines, and inflammatory mediators. In turn, these immunosuppressive cells provide a rich proangiogenic and pro-tumoral microenvironment, and prevent the innate immunity and T-cell anti-tumor immunity

The immune system is known to broadly participate in cancer-related inflammation that could precede the development of malignancy or be induced by oncogenic changes, thus generating a pro-tumor inflammatory environment.^9^ In this section we retrospectively present the relationship of the innate and adaptive immune system during response to inflammation with tumor initiation and progression and discuss the outstanding questions that remain to be answered (Fig. 3).

### Cancer-promoting inflammation

Inflammation has been recognized closely involved in cancer, substantially contributing to the development and progression of malignancies.^253^ Chronic inflammation driven by immune cells and molecular signaling pathways has been reported to lead to the susceptibility of the human body to various cancers. Evidence has shown that up to 25% of cancers are related to chronic inflammatory diseases; however, the exact mechanism underlying this connection remains unclear.^254^ Certain chronic inflammatory diseases have been recognized as precancerous lesions of tumors in clinical terms. For instance, the inflammatory bowel disease (IBD) is well known as a precancerous lesion of CRC. Clinical observations have shown that IBD might evolve into malignant tumors in the span of several years to decades. Furthermore, chemical induction of IBD is known to be a classical method for induction of CRC in mice.^265,266^ IBD-associated colon cancer shows different DNA methylation level compared with sporadic colon cancers.^267^ Besides, a research using the single-cell multiomics sequencing of human CRC has revealed that epigenetic inheritance plays an important regulatory role in the occurrence and development of CRC.^268^

Some viruses and bacteria-induced chronic inflammatory diseases have also been reported to contribute to carcinogenesis. For instance, infection with *H. pylori* was demonstrated to lead to gastritis and stomach cancer.^269,270^ IBD was not sufficient to induce CRC in the absence of intestinal microbiota or microbial products,^271^ and the use of a common antimicrobial additive could increase colonic inflammation and colitis-associated colon tumorigenesis in mice.^272^

Infection with HBV was also shown to induce chronic hepatitis, which could progress to primary HCC.^4,273^ Cervical carcinoma is known to be caused by infection with HPV.^274^ Besides, microbiota are known to directly or indirectly (*via* their metabolites, such as polysaccharide β-dextran, LPS, deoxycholic acid (DCA), short-chain fatty acid (SCFA), butyrate, and propionate) affect the differentiation and function of immune cells (e.g., M2 macrophages,^275^ neutrophils,^276^ Treg cells,^277–279^ T cells,^280^ and NKT cells^281^), potentially altering their effects on cancer. Therapies targeting gut microbiota showed significant improvement on immunotherapy efficacy.^282^

In addition, some chronic autoimmune diseases have also been associated with tumorigenesis.^283^ Moreover, in the chronic inflammatory microenvironment, a large number of immunosuppressive cells inhibit the killing function of T cells and lead to immune escape, thus promoting tumor formation. Evidence has suggested that chronic inflammatory stimuli increase the risk of cancer, promote tumor progression, and support metastatic spread.^253^ Thus, inflammatory cells and cytokines during chronic inflammation might act as tumor promoters affecting cell survival, proliferation, invasion, and angiogenesis. Here we focus on the role of the activated innate and adaptive immunity, as well as systemic inflammation in promoting cancer.

#### Cancer-promoting systemic inflammation

Systemic inflammation is a cardinal characteristics of malignancy, and it is well-established that patients with systemic inflammation always have poorer outcomes.^284^ It has been indicated that low-grade systemic inflammation related to obesity or depression promotes the progression of cancer by remodeling of the immune cell landscape.^285,286^

Obesity, characterized by chronic and low-grade systemic inflammation, increases the risk of many cancer types, such as breast cancer,^287^ CRC,^288^ liver cancer,^289^, and ovarian cancer,^290^ is associated with poor outcomes.^291^ Obesity-associated inflammation is always triggered by excessive nutrients, and is primarily localized in specialized metabolic tissues such as white adipose tissue.^292^ Tumor cell biology is directly affected by multiple cellular players present in the adipose tissue microenvironment, which have diverse morphologies and play opposing functions.^293^ White adipose tissues secrete a variety of inflammatory molecules that can potently fuel cancer, such as TNF-α, IL-6, IL-1β, and CCL2.^294,295^ These cytokines can establish chronic inflammatory environment by recruiting lymphocytes and macrophages. For example, cancer-associated adipocytes can facilitate radio-resistance by secreting IL-6.^296^ However, brown adipose tissue possesses a therapeutic potential role against cancer. The activation of the brown adipose metabolism can improve insulin resistance, reduce inflammation and increase the secretion of anti-inflammatory molecules, creating an anti-tumorigenic microenvironment.^297^ On the other hand, during the process of white adipocytes transdifferentiate into pink adipocytes in breast tissue, mammary epithelial secretory cells will lose the expression of peroxisome proliferator-activated receptor (PPAR)-γ, resulting in a pro-tumorigenic microenvironment.^298^ Given the key role of inflammation in obesity-associated cancers,^299,300^ anti-inflammatory therapies in obese patient populations may be beneficial to cancer prevention and treatment.

Chronic stress triggered by depression, anxiety, or loneliness/social isolation can also cause corresponding changes in immune function and inflammatory response, which are implicated in tumorigenesis and cancer development.^286,301,302^ First, chronic stress stimulates the classical neuroendocrine system, such as hypothalamic-pituitary-adrenal (HPA) axis, and the sympathetic nervous system (SNS), and causes a dysfunction of the prefrontal cortex and the hippocampus under stress. Subsequently, stress hormones produced during the activation of HPA axis and the SNS can facilitate tumorigenesis and cancer development through a variety of mechanisms, such as inducing DNA damage, accelerating p53 degradation, and regulating the TME. Chronic stress can also activate the inflammatory response, and the interaction between inflammatory cells and tumor cells to form the inflammatory TME, and promote tumorigenesis.^303,304^ Moreover, chronic stress can also selectively suppress the CTLs-mediated cellular immunity and interferon production, and dampen immune surveillance, thereby increase the risks of metastasis and decrease the effectiveness of anti-tumor therapy.^305,306^

In consideration of a long-term inflammatory response and the decline of the immune surveillance capabilities are implicated in tumorigenesis and cancer development,^4^ clinical management of systemic inflammation is essential for prevention and treatment of cancer.

#### Cancer-promoting inflammation in innate immunity

The innate immune response is the non-specific defense function that is formed during the development and evolution of lineage after birth.^249,307^ Innate immune cells including NK cells, macrophages, neutrophils, DCs, and innate lymphoid cells (ILCs), are known to be involved in the initial response to tissue injury and can promote or prevent tumor initiation and progression.^4^ Meanwhile, they have also been reported to facilitate cellular transformation and malignant development. Understanding the mechanism by which the innate immune system affects cancer formation and progression is crucial for developing strategies to treat cancer. In addition, other innate immune cells, such as mast cells, and MDSCs found in the TME are also involved in cancer promotion.^5^

Inflammation is often accompanied by the recruitment of fibroblasts and the induction of fibrosis. Cancer-associated fibroblast (CAFs) are responsible for the deposition of collagen and various ECM components in the TME, where they have been shown to facilitate cancer cell proliferation and angiogenesis.^308,309^ Moreover, CAFs are also known to have a critical immune function, as they produce numerous cytokines and chemokines, including osteopontin, CXCL1, CXCL2, CXCL12, CXCL13, IL-6, IL-1β, and CCL-5.^310,311^ It has been reported that during tumorigenesis, fibroblasts sense the alterations in tissue architecture caused by the increased proliferation of neighboring epithelial cells, and respond to these changes by producing pro-inflammatory mediators.^312^ In addition, CAFs have also been found to be activated during therapy-induced hypoxia, producing abundant TGF-β and numerous chemokines, including CXCL13.^313^ Subsequently, the CAF-secreted TGF-β inhibits the activation of NK cells and CTLs, and suppresses the differentiation of Treg cells and immunosuppressive plasmocytes.^314,315^ Besides, CAF-secreted CXCL13 was demonstrated to mediate the recruitment of B cells into androgen-deprived prostate cancer, resulting in hormone resistance.^313,316^ In breast cancer, the CAF-secreted CCL2 was shown to lead to the recruitment of macrophages to the TME.^317^ Furthermore, activated CAFs expressing the fibroblast activation protein-α (FAP) were also reported to attenuate anti-tumor immunity in established Lewis lung carcinoma mouse model.^318^

As tissue-resident sentinel cells, mast cells are first in the line of defense among innate immune cells responding to allergens, pathogens, or other pro-inflammatory and toxic agents.^319^ Upon activation, mast cells were found to not only rapidly release a series of biologically active mediators stored in their cytoplasmic granules, such as histamine, serotonin, TNF-α, proteoglycans, and various proteases, but could also release *de novo* synthesized lipid mediators (e.g., prostaglandins and leukotrienes), cytokines, chemokines, leukotrienes, and growth factors.^320^ In turn, many mast cell-released mediators, such as IL-1β, IL-6, TNF-α, PGE_2_, LTB_4_, and leukotriene D4 (LTD_4_), can attract or activate other immune, endothelial, epithelial, neuronal, and stromal cells. Accumulation of DCs has been observed in inflammatory diseases and multiple types of cancer, such as CRC, prostate cancer, pancreatic adenocarcinomas, esophagus squamous cell carcinomas (ESCC), non-small-cell lung cancer (NSCLC), and several types of hematologic neoplasms, such as non-Hodgson’s lymphoma, and follicular lymphoma.^321–323^ Furthermore, high density of mast cells was shown to be predictive of poor clinical outcome in CRC, lung cancer, and pancreatic cancer.^324–327^ Evidence have shown that mouse mast cells highly express programmed death ligand-1 (PD-L1) and PD-L2,^328^ indicating an additional mast cell-driven mechanism enhancing the pro-tumorigenic effect of the programmed death-1 (PD-1)/PD-L1 axis. Collectively, through shaping an inflammatory TME for immune escape, mast cells have been suggested to promote tumor development and progression. Mast cells were reported to promote the growth of endothelial cells and angiogenesis by either producing heparin or releasing lysozyme to dissolve the surrounding stromal tissue and then promote tumor growth and metastasis.^319,320^ Certain substances in the granular composition of mast cells could also promote collagen lysozyme produced by fibroblasts and tumor cells, and indirectly caused the disintegration of collagen, thus promoting tumor invasion and metastasis.^329^

Tumor-associated macrophages (TAMs), mainly M2-type macrophages are known to inhibit the killing function of T cells, and secret cytokines to maintain the immunosuppressive state in the TME, thus acting as a paradigm for tumor-promoting inflammation.^330,331^ In addition, M2-TAMs were found to regulate the distortion of adaptive responses, angiogenesis, cell proliferation, deposition, and remodeling of stromal cells in the TME.^332^ The functional reprogramming of TAMs was shown to be orchestrated by stimulations and signals from cancer cells, T and B cells. In general, the M2-like properties of TAMs resemble those of immune-tolerant macrophages, although there are multifarious phenotypes and signaling pathways in different tumors.^332^

Neutrophils act as the first line of defense of the body against infection and have been shown to respond to diverse inflammatory stimulation, with their persistent infiltration being a hallmark of chronic inflammation that contributes to tissue damage.^333,334^ They are regarded as “kamikaze” cells, sacrificing themselves while killing invading pathogens through the employment of multiple mechanisms: phagocytosis, secretion of ROS, hyperchlorous acid, and antimicrobial proteins (e.g., defensin, lysozyme, elastase, and cathepsin), or extrusion of DNA to generate NETs. In addition to playing tumor-promoting roles in the context of innate immune inflammation and tumor initiation, tumor-associated neutrophils (TANs) have also been reported to promote tumor progression by suppressing the function of the adaptive immune response in the TME.^335–337^ The increase of TANs has been found that negatively correlated with severe disease and poor outcome of patients in a broad variety of neoplasias.^338^ Similar to TAMs, TANs are also classified into N1 anti-tumor and N2 pro-tumor subsets, with neutrophil polarization influencing the role they play in the TME.^308^ N2 neutrophils were found to induce the switch of tumor angiogenesis during early tumor promotion, remodel the extracellular matrix of TME to promote tumor cell growth, regulate the biological behaviors of tumor cells, maintain the immunosuppressive state in the microenvironment by secreting various cytokines (e.g., iNOS, VEGF, Arg-1, CCL17, PGE_2_, and B/MMP9 gelatinase), and promote the invasion and metastasis of tumor cells at the later stage.^339^

Eosinophils, which are characterized by large secretory granules within their cytoplasm, are known to regulate the immune response through the presentation of antigens to T cells and release of immunomodulatory molecules.^340^ Responding to diverse stimuli, they have been reported to migrate to sites of inflammation, synthesizing and secreting several immunomodulatory molecules, including granule proteins that could potentially kill tumor cells.^103^ Alternatively, eosinophils could secrete both proangiogenic and matrix-remodeling soluble mediators that could facilitate tumor progression. Tumor-associated tissue eosinophilia (TATE) has been observed in many hematological and solid malignancies (e.g., colon, breast, colorectal, nasopharyngeal, oral, gastric, and head and neck cancers) with a generally good prognostic value,^341^ suggesting the involvement of eosinophils in the anti-tumor response. In these types of cancers, eosinophils were observed to display cytotoxicity *via* the secretion of granule proteins, TNF-α, and granzyme A,^342^ and shaped the TME *via* the induction of CD8^+^ T cells, promotion of vascular normalization, and shifting of TAMs into a pro-inflammatory (M1) phenotype.^343^ It was also found that IL-10- and IL-12-activated eosinophils suppressed the growth of prostate cancer cells in vitro and upregulated the expression of adhesion molecules, potentially limiting cancer metastasis.^344^ However, TATE has also been associated with poor prognosis in Hodgkin lymphoma and oral squamous cell carcinomas (OSCCs).^345,346^ Besides, tumor cell-derived thymic stromal lymphopoietin (TSLP) was shown to facilitate proliferation, increase the production of anti-inflammatory cytokines (e.g., IL-10, IL-4, IL-5, and IL-13), and decrease the expression of CD80 and CD86 in eosinophils, thus enhancing the proliferation of cervical cancer cells. Eosinophils were demonstrated to promote tumor metastasis and angiogenesis *via* the secretion of MMP9, VEGF, FGF, and PDGF, while polarize TAMs into a pro-tumor (M2) phenotype *via* the production of IL-4 and IL-13.^347^ Thus, the function of eosinophils might depend on the cellular composition of the TME in different cancer types.

The accumulation of MDSCs in peripheral tissues in cancer is well known along with their pro-tumor role in tumor progression. More specifically, MDSCs are known to produce Arg-1, iNOS, IL-10, TGF-β, and COX-2 to inhibit the proliferation and function of T cells.^348^ In addition to their immune suppressive function, MDSCs were shown to promote tumor progression by remodeling of the TME and facilitated tumor angiogenesis by producing cytokines, such as VEGF and FGF.^349^ In addition, MDSCs were observed to participate in the formation of premetastatic lesions, and metastasis by infiltrating primary tumors. They inhibited cellular senescence in spontaneous prostate cancer by antagonizing the IL-1α signaling pathway. Moreover, the recruitment of g-MDSCs promoted IBD and contributed to the initiation and development of CRC.^350^

#### Cancer-promoting inflammation in adaptive immunity

The adaptive immune response which occurs after the innate immune response, is a specific response of lymphocytes to antigen stimulation, followed by the immune memory effect.^351^ When antigen-presenting cells (APCs) present antigens to T cells, the T-cell receptor (TCR) recognizes the antigen and activates the secretion of tumor-killer molecules, such as IFN-γ and granzymes with the action of synergistic stimulatory molecules. Meanwhile, helper T cells secrete cytokines to activate B cells, which produce antibodies, mediating the ADCC.^249,352^ Generally, adaptive immune responses are known to inhibit tumorigenesis and progression. However, some types of T cells have been shown to mainly participate in adaptive immune responses, promoting tumor progression. In fact, Th2, Th17, and Treg cells have often been associated with tumor progression and unfavorable prognosis.^249^

T-helper 2 (Th2) cells are known to not only regulate protective type 2 immune responses to extracellular pathogens, such as helminthes, but also contribute to chronic inflammatory diseases including asthma, allergy, as well as cancer. Increasing evidence have demonstrated a crucial role of Th2 cells in orchestrating the progress and metastasis of tumors.^353^ In addition, Th2 cells and their cytokines were shown to construct an inflammatory TME involving M2-TAMs and promote tumor metastasis in breast cancer.^354^ For example, Th2 cells are known to produce IL-4, IL-5, and IL-13, and hence are able to regulate immunity. High levels of Th2 cell-derived cytokines were detected in tumor sites of patients with breast cancer, with the levels of IL-4 and the amount of tumor-infiltrating CD4^+^ T cells being positively correlated with tumor progression, as well as with metastasis to sentinel lymph nodes,^15,355,356^ highlighting the clinical relevance of Th2 cells in the pathogenesis of breast tumors. Through the secretion of IL-4, Th2 cells were also shown to regulate the polarization and function of M2 macrophages in the TME.

Th17 cells are a specific subset of T-helper lymphocytes characterized by the high production of IL-17. Th17 cells have been associated with tumor prognosis. More specifically, Th17 cells have been reported to promote tumor growth by inducing angiogenesis and exerting immunosuppressive functions. In contrast, Th17 cells were also demonstrated to recruit immune cells into tumors, activate effector CD8^+^ T cells, directly convert them toward the Th1 phenotype, and produce IFN-γ to kill tumor cells.^357^ Moreover, specific IL-17^+^ γδT-cell subsets were observed to play an unexpected role in driving tumor development and progression.^358^ They induce an immunosuppressive microenvironment and promote angiogenesis by producing various cytokines as regulatory Th17/Treg/Th2-like cells.^358^ Moreover, these pro-tumoral IL-17+γδ-T cells can suppress the maturation and function of DCs and subsequently inhibit the anti-tumor adaptive immunity by the PD-1/PD-L1 pathway.^358–360^

Studies have revealed that Treg cells could inhibit the maturation of DCs, as well as block the phagocytosis of tumor cells and the expansion of CTLs, which leads to immune surveillance and tumor progression.^361^ Treg cells were shown to promote the development and progression of tumors by inhibiting the anti-tumor immunity in TME. In particular, Treg cells were reported to lead to immune suppression by inhibiting co-stimulatory signals by CD80 and CD86 through the cytotoxic T-lymphocyte antigen-4 (CTLA-4), secreting inhibitory cytokines, and directly killing effector T cells.^362^ Treg cells have been shown to be chemoattracted to the TME by chemokines, such as chemokine receptors (CCR4)-CCL17/22 and CXCR3-CCL9/10/11, where they are activated to inhibit anti-tumor immune responses.^363^ Indeed, a high accumulation of Treg cells in various types of cancer is associated with poor survival.^364^

Regulatory B (Breg) cells represent a subset of B cells with immunosuppressive properties.^365^ According to the expression of tumor surface markers, the production of soluble factors, and the characteristics of promoting tumor growth, a variety of human and animal tumors of the Breg subtype have been identified. Although the phenotypic markers of different tumors have been reported to be different, the typical phenotypes of both human and mouse were shown to be concentrated in memory CD27^+^ and transitional CD38^+^ B cells, exhibiting the same phenotype as plasma cells (e.g., IgA^+^CD138^+^ and IgM^+^CD147^+^).^366,367^ In both human and mouse studies, Breg cells were observed to exhibit their specific immunosuppressive effects through the secretion of cytokines, such as IL-10 and TGF-β, or through the upregulation of immunomodulatory ligands, such as PD-L1 and CTLA-4, which could attenuate the response of T and NK cells and enhance the pro-tumor effect of Treg cells, MDSCs, and TAMs.^368^

### Cancer-inhibiting inflammation

Although chronic inflammation might lead to tumorigenesis, most inflammatory cells are known to kill pathogens, promote tissue repair, and inhibit tumor growth. Both innate and adaptive immune responses inhibit tumor initiation and progression.^369^ More specifically, the immune system can recognize and destroy nascent tumor cells in a process called cancer immune surveillance, which plays an important role in cancer prevention.^15^ Recently, data obtained from a large number of studies in murine models and patients with cancer provided convincing evidence that specific innate and adaptive immune cell types, effector molecules, and pathways could sometimes work together as endogenous tumor suppression factors.^362^ However, in many cases, tumor-associated inflammation, mainly supported by innate immune cells, was reported to contribute to tumor growth. The initial innate activation is known to trigger the secretion of inflammatory, regenerative, and anti-inflammatory cytokines, subsequently activating an adaptive immune response to tumors.

#### Cancer-inhibiting inflammation in innate immunity

DCs are professional APCs linking the innate and adaptive immune system.^370^ Typically, DCs recognize a wide range of “danger signals” both from invading microbes and injured host cells through binding either PAMPs or damage-associated molecular patterns (DAMPs) to PRRs.^371^ For instance, the activation of TLRs in DCs has been shown to trigger a rapid inflammatory response to pathogens;^372^ with the presentation of tumor-associated antigens (TAA) by DCs being necessary for T-cell-mediated cancer immunity.^373^ Furthermore, DCs can also regulate immune responses by generating both central and peripheral tolerance and controlling inflammatory responses *via* multiple mechanisms, such as triggering apoptosis of autoreactive T cells and T-cell anergy, expanding Treg cells, and limiting other effector cell responses.^374^ Moreover, DCs were reported to control malignant development of colitis-associated colorectal cancer (CAC) through the production of IL-22BP, which neutralized the effect of IL-22,^375^ while IL-15-cultured DCs were shown to possess the capacity to enhance the anti-tumoral functions of γδT cells.^376^ However, it has been found that cancer cells could hijack DCs to promote chronic inflammation and prevent TAA presentation, thus accelerating tumor development. For example, TME-derived factors (e.g., IL-6 and M-CSF), as well as intracellular signaling proteins of DCs, including STAT transcription factors were demonstrated to switch the differentiation of monocytes to macrophages rather than DCs, preventing the priming of tumor-specific T cells.^377,378^ In addition, inflammatory DCs (infDCs), a subpopulation of DCs only forming in response to inflammatory stimuli, are critical to the anti-tumor immune response.^379^ In particular, infDCs were shown to migrate to lymphoid nodes and present antigens to naive CD4^+^ T cells to induce the differentiation of Th1,^380^ Th2,^381^ or Th17^382^ cells depending on the inflammatory environment. Therefore, inflammatory DCs appear not only during pathogenic inflammation, but also in experimental models of inflammatory diseases, such as in patients with rheumatoid arthritis or cancer.^383^ In summary, DCs have the potential to promote efficient anti-tumor immunity by recruiting and activating various immune cells. However, the TME is rich in immunosuppresive factors (e.g., VEGF, IL-6, PGE_2_, and IL-10) that suppress the immunostimulatory capacity of DCs and instead shift DCs into an anti-inflammatory phenotype.^384^ Nowadays, modulating the function of DCs to improve cancer immunotherapy is of particular research interest.^384^

TAMs have a dominant role as orchestrators of cancer-related inflammation. In nascent tumors, TAMs are known to display a pro-inflammatory phenotype (M1), eliminating some immunogenic tumor cells by promoting a Th1 response.^385^ Furthermore, M1-polarized macrophages are characterized by the high production of pro-inflammatory cytokines (e.g., TNF-α, IL-1β, IL-6, IL-12, CXCL9, and CXCL10), and NOS and ROS intermediates, high expression of major histocompatability complex class II (MHC-II) and co-stimulatory molecules, efficient antigen presentation, but low expression of IL-10 and arginase.^331,386,387^ Through the secretion of pro-inflammatory cytokines and chemokines, such as IL-12, CXCL9, and CXCL10, M1-macrophages have been shown to drive the polarization and recruitment of Th1 cells, thereby amplifying a type 1 response, mediating phagocytosis of intracellular pathogens and tumor cells, and eliciting tissue-disruptive reactions.^388^ Several M1 stimuli, such as LPS and IFN-γ signals can polarize macrophages toward the M1 phenotype. For example, M1-like macrophages polarized with IFN-γ and exhibiting anti-tumor activity are usually characterized by high expression of HLA-DR (MHC-II),^389^ while activation of TLR9 by CpG, plus anti-IL-10 receptor antibodies were shown to redirect TAMs from an M2 to an M1 phenotype in vivo, leading to innate response debulking large tumors.^390^ Therefore, repolarization of macrophages from a pro-tumor phenotype (M2) to cytotoxic anti-tumor effectors (M1) is expected to improve the TME and contribute to anti-tumor immunotherapy.

Contrary to the N2 phenotype, tumor-inhibitory N1-TANs have been reported to produce multiple pro-inflammatory molecules (e.g., CCL2, CCL3, CXCL8, IL-6, TNF-α, and IFN-γ), and recruit other immune cells, including DCs and CD8^+^ T cells to the tumor sites.^391^ Moreover, N1-TANs can activate CD4^+^ and CD8^+^ T cells *via* the high expression of co-stimulatory molecules (e.g., CD86, ICAM-1, OX40L, and 4–1BBL), further potentiating anti-tumor immune responses.^392^ Furthermore, studies have shown that the strong anti-tumor properties of N1-TANs were also associated with the induction of ADCC, release of cytotoxic ROS, production of NETs, and their function as APCs.^333,393,394^

The roles of other innate lymphoid cells, such as ILC1, ILC2, and ILC3 subsets, in tumors are also of interest.^395^ The formal identification of ILCs increased our understanding of their tissue distribution and established the essential functions of ILCs in diverse physiological processes. These included the resistance to pathogens, regulation of autoimmune inflammation, tissue remodeling, as well as cancer and metabolic homeostasis.^271^ Briefly, ILCs are known to be major producers of cytokines in response to tissue damage and important regulators of the inflammatory response. However, their specific roles in cellular transformation and malignant progression remain largely unknown. Hence, unraveling the role of ILCs in cancer development and the interplay between ILCs and other immune cells would significantly improve the understanding of the mechanism by which the innate immune system tunes the inflammatory response in cancer. Notably, many ILC functions appear to be regulated by mechanisms distinct from those of other innate and adaptive immune cells.^396^ The ILC family subtypes are characterized primarily by their signature cytokine secretion profiles. For instance, ILC1 produces IFN-γ, whereas ILC2 produces IL-5 and IL-13, and ILC3 produces IL-17 and IL-22. Experimental evidences from murine models and patient-based studies have elucidated the effects of ILCs on the maintenance of tissue homeostasis and the associated consequences for health and disease.^397^

Natural killer cells are prototypes of innate lymphocytes with a strong cytolytic function, fulfilling their role in host defense against microbial infection and tumors.^379^ Based on their role in inhibiting microbial infections and tumor progression, NK cells are now considered to be an important component of the immune system. Moreover, conventional NK cells have been grouped as cytotoxic, IFN-γ-producing ILCs among the emerging population of ILCs.^396^ Upon activation, NK cells have been shown to induce target cell apoptosis *via* either the release of perforin and granzymes or the expression of Fas-L and TRAIL on their surface.^398,399^ Besides their cytotoxic capacity, NK cells can secrete various cytokines, chemokines, and growth factors, including IFN-γ, IL-13, TNF, FLT3L, CCL3, CCL4, CCL5, lymphotactin (XCL1), and GM-CSF.^400^ In addition, NK cells are presumed to bridge innate and adaptive immunity through the secretion of IFN-γ, which enhances the expression of MHC I on tumor cells and the expression of MHC-II on APCs, including monocytes, macrophages, and DCs.^400^ Impaired NK cells or NK cell deficiency have been associated with an increased incidence of various types of cancer.^395,396^ Thus, NK cells are considered to be key effectors in cancer immunosurveillance, transplantation rejection, as well as early viral immunity.^400^

Interestingly, NK-T cells, expressing both the TCR of T cells and natural killer cell receptor (NKR)-P1 receptors of NK cells, play an important role in innate immunity through the recognition of lipid antigens presented by CD1d.^401^ In addition, NK-T cells are known to not only secrete Th1- and Th2-related cytokines, but also have the same killing target cells as CD8^+^ killer T cells.^401^ The activation of NK-T cells has been shown to be usually accompanied by the activation of T cells, B cells, and NK cells, therefore having a great impact on the immune response after activation.^395,402^

Although in some cases, IL-17-producing γδ-T cells were demonstrated to promote tumor growth through the production of IL-17,^403^ they are generally considered as anti-tumor innate immune cells that provide IFN-γ-mediated protective responses in certain cases. Briefly, γδ-T cells recognize and directly kill tumor cells through TCR and natural killer cell receptors (NKR).^404^ The tumor cell killing of γδ-T cells was shown to be mediated by the TRAIL associated with TNF, Fas-L, or granulosa cell pathway (leading to the secretion of perforin and granzyme).^405^ Moreover, γδ-T cells could also destroy tumor cells through ADCC after treatment with tumor-specific antibodies.^406^

Anti-inflammatory metabolites produced by immune cells have also been reported to play an important role in orchestrating the resolution of inflammation. As mentioned, SPM, such as resolvins, protectins, lipoxins, and maresins, are a family of endogenous lipid mediators that exert proresolving and anti-inflammatory effects without suppressing the immune response.^143^ During the onset phase of inflammation, macrophages secrete SPM, promoting the resolution of inflammation, and increasing vascular permeability, thus enabling the infiltration of PMN into inflammatory tissues. Moreover, SPM have been found to stimulate self-limited innate responses, enhance innate microbial killing and clearance, hence avoiding the transition to chronic inflammation and inhibiting cancer progression.^407^ Accumulating evidence have shown that SPM not only possess anti-tumor ability, but also enhance the effects of other anti-tumor therapies. For instance, low dose of LXA_4_ could inhibit the proliferation and metastasis in OVC cells, Hela cells, as well as in papilloma.^350,408,409^ Besides, RvD1 and RvD2 were shown to prevent metastasis of A549 by reducing EMT induced by TGF-β1.^410^ Likewise, RvD1 was reported to induce caspase-3, thus increasing the apoptosis of pancreatic ductal adenocarcinoma cells in vitro.^411^ Furthermore, SPM are also known to display anti-tumor actions by targeting immune cells. For instance, an LXA_4_ isomer could inhibit the infiltration of neutrophils and reduced the production of pro-inflammatory cytokines, including TNF-α, IL-1β, and IL-6, thereby inhibiting the severity of colitis in mice.^412^ In tumor-bearing mice, LXA_4_ significantly inhibited tumor growth by targeting IL-10-producing Breg cells;^26^ and selectively converted M2-TAMs to the M1 phenotype, triggering tumor cell apoptosis and thus attenuating tumor progression.^26,412^ Likewise, RvD1 was observed to enhance the killing function of NK cells in pancreatic ductal adenocarcinoma.^411^ Moreover, SPM have been reported to prevent tumorigenesis by targeting precancerous lesions. For instance, RvE1 could increase the survival rate and promote the regression of colitis.^413^ Similarly, the inhibitory effect of MaR1 in mice colitis was recently reported.^414^ In conclusion, SPM were demonstrated to play an important role in attenuating tumor-promoting inflammation, suppressing tumor development and progression, as well as enhancing anti-tumor immunity.

#### Cancer-inhibiting inflammation in adaptive immunity

Similar to APCs, macrophages and DCs also bridge innate and specific immunity.^249,352,415^ They can recognize tumor cell antigens and present them to the specialized members of the immune system to activate tumor-specific T cells for the killing and clearance of tumor cells. Adaptive immunity plays the most important role in the anti-tumor immune response.^416^ It has been noted that CD4^+^ Th1 cells, activated CD8^+^ T cells, and γδT cells are often involved in immune responses and have been associated with favorable prognosis in patients with lung cancer.^417^ In particular, CTLs are known to recognize the abnormal antigens of tumor cells, secrete granzyme and perforin to kill tumors, and express Fas-L, allowing them to bind with tumor cells to promote their apoptosis. Therapeutic reinvigoration with tumor-specific T cells has greatly improved the clinical outcome in many cancers. Nevertheless, many patients did not achieve a durable benefit. Recent evidence from studies in murine and human cancer have suggested that intratumoral T cells display a broad spectrum of dysfunctional states, shaped by the multifaceted suppressive signals occurring within the TME.^418^ However, this dysfunction of T cells in cancer might be utilized to develop personalized strategies to restore anti-tumor immunity. One such example is helper T cells that secrete cytokines, recruit, and activate CTLs to kill tumor cells.^419^

Th1 cells, a lineage of CD4^+^ effector T cells characterized by the secretion of IL-2, IFN-γ, TNF-α, and lymphotoxin are principally responsible for activating and regulating the development and persistence of CTLs. For example, Th1 cells have been shown to release IFN-γ, which stimulates the upregulation of molecules, such as LMP2, LMP7, MECL, PA28, and MHC class I in APCs, all of which contribute to increased antigen presentation to CTLs.^420^ Besides, Th1 cells are also known to recruit and activate inflammatory cells (macrophages, DCs, eosinophils, and NK cells) in the tumor, thereby enhancing their ability to eliminate intracellular microbes and to present antigens to CD8^+^ CTLs.^421^ In addition, Th1 cells were also found to directly destroy tumor cells *via* the release of cytokines, such as lymphotoxin, which activate death receptors on the surface of cancer cells.^422^ Furthermore, Th1 cells can directly interact with tumor cells through MHC class II molecules.^423^

B cells and humoral immunity have also been described to regulate anti-tumor immunity through other mechanisms, either by expressing cytokines, such as IL-10 or IL-35, inducing antibody-mediated cytotoxicity through NK cells, or by activating the C5a or C3a complement system components, which seem to either activate or suppress anti-tumor immunity in a context-dependent manner.^424^ However, the ways by which different B-cell types manifest their immunosuppressive effects remain poorly understood. Some studies have shown that B cells might play a pro-tumor role due to their immunosuppressive subtypes. For instance, tumor-infiltrating B-lymphocytes (TIBs) have been detected in all stages of lung cancer.^425^ The existence of TIBs has been reported to vary in different stages and histological subtypes, suggesting a critical role for B cells during lung cancer progression.^426^ Of interest, activated B cells were also found to be able to directly lyse tumor cells. Moreover, TIBs have been shown to possess cytotoxicity toward hepatoma cells through the secretion of granzyme B and TRAIL. In one such study, IFN-α- and TLR agonist-stimulated B cells produced functional TRAIL that was cytotoxic to melanoma cell lines.^366^ Abundant studies have assessed the function of TIL-B by immunohistochemical examination of CD20.^427^ Accordingly, 50.0% of these studies reported a positive prognostic effect for CD20^+^TIBs, whereas the rest showed neutral and negative effects. The prognostic significance of TIBs was basically reported to be consistent with that of CD3^+^ and CD8^+^ T cells, with the anti-tumor activity of T cells being generally shown to be more potent in the presence of TIB cells.^428^ Finally, accumulating evidence have supported a positive role for TIB in anti-tumor immunity,^427,429,430^ suggesting that enhancement of these responses should be considered in future cancer immunotherapies.

### Therapy-elicited inflammation

Recently, anti-cancer therapy-induced inflammation has been recognized as a strong modulator of the TME. Several conventional classes of chemotherapeutic agents (e.g., anthracyclines and oxaliplatin) and radiation therapy can elicit immunogenic cell death (ICD) of tumor cells, and induce the secretion of DAMPs from dying cells.^431–433^ Subsequently, the ICD-induced DAMPs activate DC-mediated anti-tumor T-cell responses. In fact, the host immune response is indispensable for the therapeutic efficacy of these drugs.^433^

Chemotherapeutic drugs, also known as cytotoxic drugs, display anti-cancer effects by acting on key cellular biological events necessary for the proliferation and survival of cancer cells. Besides, chemotherapy can activate immune responses and enhance the activation of effector T cells, disrupting the immunosuppressive pathway of TME.^434,435^ However, chemotherapy resistance is one of the main factors that limit the therapeutic effect and affect the clinical outcome.^2,436^ Some chemotherapeutic drugs cause inflammation events, which play a pivotal role in tumor angiogenesis, metastasis, and failure of therapy.^437^ For instance, cisplatin is one of the most effective anti-cancer drugs used to treat a variety of solid tumors.^438^ Cisplatin-induced inflammation is mediated through multiple mechanisms including activating NF-κB, COX-2, and TNF-α.^438,439^ Furthermore, celecoxib, a specific COX2 inhibitor, increase the anti-tumor efficacy of cisplatin in cervix cancer cells, as well as in bladder cancer and gastric cancer.^440–442^ Paclitaxel induces apoptosis by stabilizing microtubules, thereby leading to cell arrest.^442,443^ In response to paclitaxel administration, a variety of inflammatory factors and signaling pathways, such as IL-1β, IL-8, IL-6, and NF-κB, can be activated.^444–447^

By using high doses of radiation to kill cancer cells and shrink tumors, radiotherapy is an important approach of cancer treatment. More than half of the cancer patients receive radiotherapy during their therapies.^448^ Radiation activates the interconnected network of cytokines, adhesion molecules, ROS/RNS and DAMPs, resulting in a self-amplified cascade, which generates pro-inflammatory TME, ultimately leads to tumor cell death.^8,449^ On the one hand, inflammation triggered by radiation feeds into adaptive antigen-specific immune responses and adds another dimension to the tumor-host crosstalk during radiotherapy, which can contribute to cancer cure.^249,450^ On the other hand, radiotherapy-induced chronic inflammation in the TME causes an increase in immunosuppressive populations, such as M2 macrophages, MDSCs, and Tregs.^451^ For instance, radiation can induce IL-6/STAT3 signaling pathway, which promotes tumor invasion and facilitates the survival of tumor cells after therapy, thereby conferring resistance to therapy.^452,453^ Moreover, silencing IL-6 by siRNA inhibits tumor recurrence after radiotherapy in prostate cancer and sensitizes tumor cells to radiation.^454^

Growing evidence suggests that TME is one of the major obstacles for cancer immunotherapy, where chronic inflammation plays a predominant role in tumor cell proliferation, angiogenesis, and immunosuppression.^6,455^ Furthermore, the side-effects of immune checkpoint blockade (ICB) and CAR-T therapies, such as coagulopathy and “cytokine storm” have limited their full application in cancer therapy,^29,30,456^ suggesting that reduction of these harmful immunotherapy-associated inflammation would be beneficial for the outcome of cancer patients. However, acute inflammation induced by other therapies can improve the effectiveness of immunotherapy. For example, activation of type 1 IFN response, such as recombinant IFN, CpG oligodeoxynucleotide, and 3′3′-Cgamp, can boost anti-cancer efficacy in synergy with immunotherapies.^457^ Besides, radiotherapy can trigger acute local inflammation, which sensitizing tumor cells to ICB therapy.^458^

Hence, acute inflammation triggered by some therapies can re-educate the pro-tumor TME toward an anti-tumor immune milieu. However, it is noteworthy that chronic death/injury-induced inflammation potentially promotes tumor progression and confers resistance to therapy,^459^ implying that therapy-elicited inflammation is a “double-edged sword” for cancer.

Understanding the means by which the immune system affects cancer development and progression has been one of the most challenging questions in anti-tumor therapy. Aforementioned, chronic inflammation shapes the TME, thereby promoting tumor development and progression, whereas acute inflammatory reactions can be used to improve the efficiency of anti-tumor therapies. Given its crucial role in cancer development, progression, and the anti-tumor effects of therapy, harnessing inflammation will open up new possibilities for long-lasting, multilayered tumor control.

## Cancer therapy by targeting inflammation signaling pathways

Chronic inflammation is considered to be one of the characteristics of tumor initiation and progression,^5^ and therapy-induced chronic inflammation often endows residual cancer cells with resistance to subsequent courses of treatment (e.g., chemotherapy resistance and radiotherapy resistance).^4^ Anti-inflammatory drugs have been proven efficient for the prevention and treatment of tumors. However, the side-effects of ICB and CAR-T therapies, such as coagulopathy and the “cytokine storm” have limited their full application to cancer therapy,^29,30^ indicating that reduction of these pernicious inflammation reactions accompanying immunotherapy will improve therapeutic efficacy. Recently, a number of therapeutic strategies to limit inflammatory cells and their products have been successfully applied in clinical or preclinical tumor models. For instance, statins significantly reduced the risk of development of multiple types of cancer by exerting anti-inflammatory and other effects.^21–23^ Similarly, neutralization of IL-17A, IL-11, or IL-22 could inhibit colonic tumorigenesis at an early stage,^460–462^ while COXs inhibitors (e.g., celecoxib and aspirin) impaired tumor growth and metastasis.^463^

**Fig. 4:**
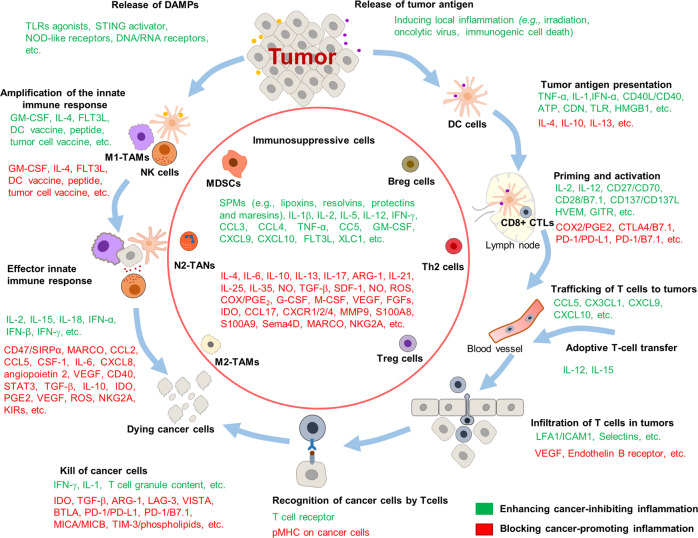
Harnessing the inflammation in cancer therapy. Several promising strategies for regulation of cancer-related inflammation have been suggested to improve anti-tumor response. On the one hand, inducing inflammation and modulating immune cell activation would overcome T-cell exclusion, turning “cold” tumors into “hot” tumors, for instance, local inflammation induced by irradiation or oncolytic viruses can promote innate immunity by activating nucleic-acid-sensing cytosolic receptors (cGAS–STING or RLRs) and subsequent type I IFN response. Besides, promoting DC maturation by cGAS–STING, TLR agonist, DC vaccine, or injection of GM-CSF can induce an acute inflammatory response and priming of T lymphocytes, which facilitate tumor regression. On the other hand, inhibiting chronic inflammation by anti-inflammatory drugs (e.g., aspirin) or accelerating inflammation resolution by proresolving mediators (SPM, e.g., lipoxins, resolvins, protectins, and maresins) also display an overall survival benefit for anti-cancer therapy. Furthermore, limitations of the infiltration and function of immunosuppressive cells (e.g., MDSCs, Treg cells, Breg cells, and M2-TAMs) by blocking inflammatory pathways is another way to restore immune surveillance and promote anti-tumor immunity. Green represents factors that enhancing these cancer-inhibiting inflammation, while red represents factors that blocking these cancer-promoting inflammation, would be beneficial to improve the effect of anti-tumor therapy

Nevertheless, not all inflammatory diseases or persistent infections are associated with increased cancer risk. Some cases of inflammation, such as allergic diseases that display a state of constant or recurring inflammation, might be even inversely correlated with cancer progression.^464,465^ In addition, although ICB has been reported to be clinically effective in presenting a durable response to treatment in some solid tumors, most patients with cancer did not respond to treatment for a variety of reasons. Infiltrated-inflamed tumors are considered “hot” tumors containing a high number of infiltrating cytotoxic lymphocytes expressing PD-1 that usually respond well to ICB.^466^ In contrast, infiltration-excluded tumors are characterized by the accumulation of CTLs along the border of the tumor mass and a lack of infiltrating CTLs into the tumor core. These tumors are generally considered “cold” tumors with poor sensitivity to ICB.^467^ Several promising strategies have been suggested to enhance the inflammatory infiltration that would contribute to the alteration of a cold into a “hot” tumor, thus rendering it sensitive to ICB.^16^ Herein, we discuss the targeted therapeutic approaches for the regulation of cancer-related inflammation (Fig. 4).

### Non-specific agents targeting chronic inflammation

#### Non-steroidal anti-inflammatory drugs

NSAIDs are a family of agents that primarily inhibit the activity of COX enzymes and thereby suppress the synthesis of prostaglandins. Importantly, NSAIDs (including aspirin, celecoxib, and ibuprofen) use has been linked to reduced cancer risk and mortality.^6^ Aspirin, one of the most widely used anti-inflammatory drugs, has been identified as a broad-spectrum cancer-preventive agent based on multiple clinical and epidemiological studies.^468–470^ Besides, both preclinical and clinical studies have demonstrated promising results of the role of celecoxib in the treatment and prevention of cancer, and the best outcomes were observed in colon, breast, prostate, and head and neck cancers.^471^ Nevertheless, long-term administration of NSAIDs can result in side-effects including mucosal lesions, bleeding, peptic ulcer, and intestinal inflammation.^20^ Thus, the benefits of taking NASIDs for prevention and/or treatment of cancer must be assessed by risk versus benefit analyses.

#### Statins

Similar to aspirin, other agents with conventional anti-inflammatory activities have been repurposed for use in the prevention or/and treatment of cancer. Statins, a family of compounds that reduce blood cholesterol concentration by inhibiting the 3-hydroxy-3-methylglutaryl coenzyme A (HMG-CoA) reductase, have a wide spectrum of activity as anti-cancer agents, including anti-angiogenic and anti-inflammatory actions in preclinical studies.^472,473^ However, a definitive benefit of statin use has not been confirmed in a randomized controlled trial setting to date. Several trials in patients with different tumor types designed to evaluate the benefit of statin are currently ongoing (NCT: 02161822, 01821404).

#### Corticosteroids

Corticosteroids, the most effective anti-inflammatory drugs for many chronic inflammatory diseases, are also shown to have anti-cancer activity.^474^ For example, pre-treatment with dexamethasone (DEX) improves the efficacy of chemotherapy in xenograft or syngeneic experimental tumor models of glioma, breast cancer, lung cancer, and CRC.^475^ Clinical trials have demonstrated that DEX in combination with carfilzomib and lenalidomide, obviously improved progression-free survival of patients with relapsed multiple myeloma.^476,477^

#### Natural anti-inflammatory products

By reducing the production of pro-inflammatory AA metabolites, omega-3 fatty acids reduce the production of eicosanoids that can activate AP-1 and NF-kB signaling and promote angiogenesis.^474,478^ Evidence has demonstrated that omega-3 fatty acids supplementation is associated with a reduced risk of CRC among individuals with low plasma levels of such fatty acids at baseline and in the African-American population.^479^

Several natural products such as polyphenols are able to modulate NF-κB, Wnt/β-catenin, PI3K/Akt, and MAPKs signaling and prevent the occurrence of inflammatory conditions.^480^ As a source of modulating agents to suppress chronic inflammation, dietary polyphenols may function as a chemopreventive agent against cancer, and improve the therapeutic effect of cancer.^481^ For example, curcumin (diferuloylmethane) is an active ingredient in plant turmeric spices, the anti-cancer activity of curcumin is related to its activity on inhibiting NF-κB, MAPK, PI3K/Akt/mTOR, Wnt/β-catenin, and JAK2/STAT3 signaling pathways.^482,483^ Besides, resveratrol is a natural polyphenol that provides a number of anti-aging health benefits including cardioprotection, and cancer prevention;^484,485^ while many factors need to be explored before resveratrol can be applied for human cancer prevention or treatment.

### Reducing the therapy-elicited chronic inflammation

As discussed above, despite acute inflammation elicited-therapy contributes to destroy cancer cells during treatment, the therapy-elicited chronic inflammation (e.g., IL-1β, IL-6, IL-8, COX2/PGE_2_, NF-κB, and DAMP) plays a pivotal role in promoting therapeutic resistance and cancer progression.^486,487^ Scientists hypothesized that blocking chronic inflammation might enhance the therapeutic efficacy and benefit to cancer patients.^16^ For example, both chemotherapeutic drugs and radiation can induce IL-6 expression in tumor and stromal cells^452,453^ through the activation of NF-κB signaling, causing therapeutic resistance.^488,489^ These evidences suggest that blocking IL-6 or it’s downstream signaling pathways may provide therapeutic enhancement. Nowadays, several trials designed to evaluate the efficacy of Tocilizumab (human IL-6R-specific antibody) in chemotherapy are ongoing (Table 1).

**Table 1:** Agents targeting cancer-associated inflammation signaling pathways in ongoing or completed clinical trials

Drug name	Target	Condition	Phase	NCT number	Current status
*Non-specific agents*					
Asprin	COX-1/2	Gastric cancer	III	NCT04214990	Recruiting
		Cancer-associated thrombosis in solid tumor	I	NCT02285738	Completed
		Multiple myeloma	II	NCT01215344	Completed
		Colon cancer	III	NCT02467582	Recruiting
Celecoxib	COX-2	Primary breast cancer	III	NCT02429427	Completed
		Locally advanced NSCLC	I	NCT00046839	Completed
		Lung cancer	II	NCT00020878	Completed
		Stage II, III, and I breast cancer	II	NCT00201773	Completed
		Prostate cancer	II	NCT01220973	Completed
		Metastatic colorectal cancer	II	NCT00466505	Completed
		Head and neck cancer	I	NCT00581971	Completed
		Head and neck cancer	II	NCT00061906	Completed
		Metastatic kidney cancer	II	NCT01158534	Completed
		Cervical intraepithelial neoplasia	II	NCT00081263	Completed
		Colorectal cancer	II	NCT00033371	Completed
		Mouth neoplasms	II	NCT00953849	Completed
		Malignant peritoneal mesothelioma	II	NCT02151448	Completed
		Anaplastic glioma	II	NCT00504660	Completed
		Multiple myeloma	II	NCT00099047	Completed
		Liver cancer	III	NCT03059238	Completed
Rosuvastatin	HMG-CoA	Breast cancer	II	NCT01299038	Completed
		NSCLC	I	NCT02317016	Completed
		Advanced solid malignant tumors	I	NCT02106845	Completed
		Ovarian cancer	II	NCT03532139	Recruiting
		Rectal cancer	II	NCT02569645	Recruiting
		Squamous cell carcinoma	I	NCT00966472	Completed
		Endometrial carcinoma	II	NCT04491643	Recruiting
		Leukemia, myeloid, acute	I	NCT03720366	Recruiting
Dexamethasone	Undefined	Prostate cancer	II	NCT01036594	Completed
		Ovarian cancer	IV	NCT00817479	Completed
		Early-stage breast cancer	IV	NCT03348696	Completed
		Lung cancer	III	NCT00403065	Completed
		Hepatic cancer	II	NCT00587067	Completed
		Brain tumor	III	NCT00088166	Completed
*Cytokines and chemokines*					
Infliximab	Chimeric TNFα- antibody	Pancreatic neoplasms	II	NCT00060502	Completed
		Lung neoplasm malignant	IV	NCT04036721	Recruiting
		Melanom	II	NCT04305145	Recruiting
		Hepatosplenic T-cell lymphoma	IV	NCT01804166	Completed
		Renal cell carcinoma	IV	NCT02596035	Active, not recruiting
Etanercept	Human TNFR2–Fc fusion protein	Pancreatic neoplasms	II	NCT00201838	Completed
		Melanoma	VI	NCT01053819	Completed
		Metastatic castration-resistant prostate cancer	I	NCT03792841	Recruiting
		Leukemia	II	NCT00509600	Completed
Tocilizumab	Human IL-6R-specific antibody	Urothelial carcinoma	I/II	NCT03869190	Recruiting
		Breast cancer	I	NCT03135171	Recruiting
		Pancreatic carcinoma	II	NCT02767557	Recruiting
		Advanced liver cancers	I/II	NCT04524871	Recruiting
		Hematologic malignancy	II	NCT04395222	Recruiting
		Prostate adenocarcinoma	II	NCT03821246	Recruiting
		Colorectal cancer	I	NCT03866239	Recruiting
		Non-small-cell lung cancer	I/II	NCT03337698	Recruiting
		Diffuse large B-cell lymphoma	I/II	NCT03677154	Recruiting
Siltuximab	Chimeric anti-IL-6 antibody	Prostate cancer	II	NCT00433446	Completed
		Metastatic pancreatic adenocarcinoma	I/II	NCT04191421	Recruiting
		Prostatic neoplasms	I	NCT00401765	Completed
		Solid tumors	I/II	NCT00841191	Completed
		Multiple myeloma	II	NCT00402181	Completed
		Myeloma	I/II	NCT01531998	Completed
		Metastatic renal cell carcinoma	I/II	NCT00265135	Completed
		Lymphoma, non-Hodgkin	I	NCT00412321	Completed
Carlumab	Human anti-CCL2 antibody	Prostate cancer	II	NCT00992186	Completed
MABp1	human anti-IL-1α antibody	Advanced cancers	I	NCT01021072	Completed
Reparixin	inhibitor of CXCR1/2	Metastatic breast cancer	I	NCT02001974	Completed
		Metastatic breast cancer	II	NCT02370238	Unknown
Plerixafor	inhibitor of CXCR4	Metastatic pancreatic cancer	II	NCT04177810	Recruiting
		Hematologic neoplasms	II	NCT00914849	Completed
		Advanced pancreatic, ovarian and colorectal cancers	I	NCT02179970	Completed
		Brain tumors	I/II	NCT01288573	Completed
		Multiple myeloma	II	NCT01753453	Completed
		Hematological malignancies	I/II	NCT00241358	Completed
		Advanced cancer	I	NCT03240861	Recruiting
		Acute myeloid leukemia	I	NCT00990054	Completed
		Non-Hodgkin’s lymphoma	VI	NCT01164475	Completed
Anakinra	human recombinant IL-1 receptor antagonist	Multiple myeloma and plasma cell neoplasm	II	NCT00635154	Completed
		Breast cancer	I/II	NCT02018458	Completed
		Testis cancer	II	NCT04150848	Recruiting
		Breast cancer	II	NCT01740323	Completed
		Multiple myeloma	I/II	NCT03430011	Recruiting
		Multiple myeloma	II	NCT03233776	Completed
		Advanced malignant neoplasm	III	NCT03525873	Recruiting
		Cervical cancer	II	NCT00319748	Completed
		Recurrent or refractory large B-cell lymphoma	II	NCT04205838	Recruiting
		Advanced cancers	II	NCT00379353	Completed
*Inflammatory transcription factors*					
Ruxolitinib	JAK1/2 inhibitor	Pancreatic cancer	II	NCT01423604	Completed
		ER-positive breast cancer	II	NCT01594216	Completed
		Colorectal cancer	I	NCT04303403	Recruiting
		Myeloproliferative neoplasms	I	NCT02076191	Completed
		Head and neck squamous cell carcinoma	II	NCT03153982	Recruiting
		Acute myeloid leukemia	II	NCT00674479	Completed
		Hematopoietic neoplasm	II	NCT01523171	Completed
		Bladder cancer	II	NCT02788201	Completed
Pacritinib	JAK2 inhibitor	Acute myeloid leukemia	I	NCT02323607	Completed
		Prostate cancer	II	NCT04635059	Not yet recruiting
		Breast cancer	I/II	NCT04520269	Recruiting
		Relapsed/refractory lymphoproliferative disorders	I	NCT03601819	Recruiting
Bortezomib	NF-κB inhibitor	Prostate cancer	II	NCT00183937	Completed
		Kidney cancer	II	NCT00025376	Completed
		Lung cancer	II	NCT00064012	Completed
		Recurrent breast cancer stage IV breast cancer	II	NCT00025584	Completed
		Head and neck cancer	I	NCT00629226	Completed
		Colorectal cancer	I	NCT00280176	Completed
		Non-small cell lung cancer	II	NCT01833143	Completed
		Colorectal cancer	II	NCT00052507	Completed
		Ovarian cancer	I	NCT00098982	Completed
		Bladder cancer	II	NCT00066352	Completed
Trabectedin	TAMS cytotoxicity	Prostate cancer	II	NCT00147212	Completed
		Ovarian cancer	III	NCT00113607	Completed
		Pancreatic cancer	II	NCT01339754	Completed
		Neoplasm metastases	I/II	NCT01267084	Completed
		Solitary fibrous tumors	II	NCT03023124	Recruiting
		Solid tumor	II	NCT00786838	Completed
		Soft tissue sarcoma or Ewing’s family of tumors	II	NCT00070109	Completed
		Liposarcoma	II	NCT00060944	Completed
		Brain and central nervous system tumors	II	NCT00003939	Completed
		Recurrent high grade meningioma	II	NCT02234050	Completed
		Sarcoma	II	NCT00379145	Completed
		Advanced and/or metastatic liposarcoma	II	NCT01692496	Completed
RG7155	Human CSF-1R specific antibodies/TKIs	Solid cancers	I	NCT02323191	Completed
		Neoplasms	I	NCT02760797	Completed
		Platinum-resistant ovarian, fallopian tube, or primary peritoneal cancer	II	NCT02923739	Active, not recruiting
		Advanced solid tumors	I	NCT01494688	Completed
IMC-CS4	Human CSF-1R specific antibodies/TKIs	Advanced solid tumors	I	NCT01346358	Completed
		Breast or prostate cancer	I	NCT02265536	Completed
		Pancreatic cancer	I	NCT03153410	Recruiting
PLX3397	Human CSF-1R specific antibodies/TKIs	Metastatic breast cancer	I/II	NCT01596751	Completed
		Solid tumors	I	NCT01525602	Completed
		Gastrointestinal stromal tumors	I/II	NCT02401815	Completed
		Tenosynovial giant cell tumor	IV	NCT04526704	Recruiting
		Sarcoma	I/II	NCT02584647	Recruiting
		Prostate cancer	I	NCT02472275	Completed
		Metastatic/advanced pancreatic or colorectal cancers	I	NCT02777710	Completed
		Refractory leukemias and refractory solid tumors	I/II	NCT02390752	Recruiting
		Acute myeloid leukemia	I/II	NCT01349049	Completed
		Breast cancer	I	NCT01042379	Recruiting
		Hodgkin lymphoma	II	NCT01217229	Completed
		Glioblastoma	I/II	NCT01790503	Completed
AMG 820	Human CSF-1R specific antibodies/TKIs	Advanced solid tumor cancer	I/II	NCT02713529	Completed
		Advanced solid tumors	I	NCT01444404	Completed
CP-870	Human CD40 agonist antibody	Solid tumors	I	NCT02157831	Completed
		Adenocarcinoma pancreas	I	NCT01456585	Completed
		Metastatic melanoma	I	NCT01103635	Completed
		Completed	I	NCT0100852	Completed
Tasquinimod	MDSCs	Prostate cancer	III	NCT01234311	Completed
		Prostate cancer	I	NCT01513733	Completed
		Prostate cancer	II	NCT00560482	Completed
		Prostate cancer	II	NCT02159950	Completed
		Hepatocellular, ovarian, renal cell and gastric cancers	II	NCT01743469	Completed
		Multiple myeloma	II	NCT04405167	Recruiting
Sipuleucel-T	DC vaccine	Metastatic prostate cancer	II	NCT01560923	Completed
		Prostate cancer	II	NCT00901342	Completed
		Prostate cancer	III	NCT00779402	Completed
Ipilimumab	Anti-CTL4A mAb	Non-small cell lung cancer	II	NCT01820754	Completed
		Prostate cancer	II	NCT01194271	Completed
		Pancreatic cancer	I	NCT00836407	Completed
		Selected advanced tumor	I	NCT01750580	Completed
		Breast cancer	I	NCT01502592	Completed
		Prostate cancer	III	NCT00861614	Completed
		Metastatic colorectal cancer	III	NCT04008030	Recruiting
		Solid tumor	II	NCT03865082	Recruiting
		Gastrointestinal stromal tumor	I	NCT01643278	Recruiting
		Cervical adenocarcinoma	I	NCT01711515	Completed
Tasquinimod	S100A9-TLR-RAGE axis inhibition	Prostate cancer	III	NCT01234311	Completed
		Metastatic hormone-resistant prostate cancer	II	NCT02159950	Completed
		Hepatocellular, ovarian, renal cell and gastric cancers	II	NCT01743469	Completed
		Multiple myeloma	I	NCT04405167	Completed
BMS-936559	PD-L1-inhibiting IgG4 mAb	Cancer, multiple indications	I	NCT00729664	Completed
E7766	STING agonist	Lymphoma, advanced solid tumors	I	NCT04144140	Recruiting

Evidence demonstrates that many DAMPs are released from dying cells after chemo-/radiotherapy.^490^ In the TME, DAMPs can be ligands for TLRs expressed on immune cells and induce cytokines production and T-cell activation.^491^ However, DAMPs released from tumor cells can directly activate tumor-expressed TLRs that induce chemoresistance and metastasis.^492,493^ Furthermore, DAMP-induced chronic inflammation in the TME causes an increase in the infiltration of immunosuppressive cells, such as M2 macrophages, MDSCs, and Tregs.^494–496^ Therefore, regulation of DAMPs after chemo-/radiotherapy can reduce excessive inflammation to create an immunogenic TME.

In brief, combining educating inflammatory TME with anti-cancer therapies (including chemotherapy, radiotherapy, and immunotherapy) may provide more efficient strategy to inhibit tumor growth and improve patient survival.

### Adjusting the inflammation in innate immunity

Inflammation is classically viewed as a feature of innate immunity, which differs from adaptive immunity owing to the receptors mediating its activation and its rapid onset. Once activated by PAMPs or DAMPs, MHC class I and II, and co-stimulatory molecules, as well as numerous inflammatory chemokines and cytokines are upregulated, attracting and priming T cells for activation through diverse antigen receptors.^497,498^ Given the crucial role of innate immune responses in immunity and inflammation,^252,351^ harnessing these responses opens up new possibilities for long-lasting, multilayered tumor control (Fig. 3).

#### Targeting mast cells

There have been compelling evidence that mast cells in the network of immune cells are involved in inflammatory disease and cancer.^322,499^ A number of studies have documented that mast cells potentially facilitate tumor progression *via* enhancing tumorigenesis, angiogenesis, and tissue remodeling, as well as *via* shaping an inflammatory microenvironment for immune escape.^499–503^ Today, several therapeutic strategies have been developed to inhibit tumor growth and improve the effect of immunotherapy by targeting mast cells. For instance, such strategies include the alteration of the numbers of mast cells, suppression of their activation, and prevention of the effects of inflammatory mediators.

Reducing the infiltrating numbers of mast cells is a promising treatment approach in inflammatory disease and cancer, in which their numbers are increased. The numbers of tumor-infiltrating mast cells have been reported to be reduced by the specific induction of apoptosis or by blocking the effects of factors that promote the recruitment of mast cell progenitors, their migration, differentiation, or survival.^499^ The stem cell factor (SCF-1), *via* the activation of the c-kit receptor (CD117) expressed on mast cells, has been identified as one of the most important factors for regulating the numbers of tissue mast cells under physiological conditions.^504^ Besides, several mediators of the recruitment of mast cell progenitors, such as CCR2, CCR3, CCR5, CCL2, CXCR2, IL-4, and CXCL12, were also considered to be important for the accumulation of mast cells in affected organs in murine models of disease.^505,506^ Blocking antibodies against these mediators have been used in various animal models to attempt to reduce the numbers of infiltrating mast cells in inflammatory diseases.^507^ Importantly, blocking these signals was also shown to display a potent anti-tumor effect.

Interestingly, mast cells are known to express a substantial number of activating and inhibitory receptors, which could be exploited to interfere with their activation.^508^ Recently, several drugs, are used in the clinic to treat mast cell-driven disorders, mostly allergic diseases. These include cromolyn sodium, nedocromil, lodoxamide, and antagonists for the histamine receptor H1, such as azelastine, ketotifen, olopatidine, bilastine, desloratadine, rupatadine, and epinastine.^509^ However, whether these drug have an anti-tumor effect remains unknown.

The degranulation of mast cells, the process of releasing inflammatory mediators from secretory granules, is a consistent feature of inflammatory lesions or tumors.^509–511^ Studies have shown that PI3K plays a crucial role in the biological functions of mast cells, including degranulation.^512^ Treatment with LY294002, a specific PI3K inhibitor, or inhibition of PI3K by overexpression of the Δp85 dominant-negative inhibitor was reported to lead to a significant decrease in the degranulation of mast cells *via* antigen-induced calcium (Ca^2+^) signals.^513^ Interference of the degranulation of mast cells was also shown to suppress tumor angiogenesis and progression in Myc-induced β-cell pancreatic cancer.^514^ Moreover, IL-10 inhibited the degranulation of mast cells by suppressing the expression and signaling of the IgE receptor of mast cells,^515^ while blocking IL-10 could hinder the antigen-induced recruitment of mast cell progenitors to the lungs of C57BL/6 mice.^516^ In addition, pharmacologic inhibition of the degranulation of mast cells using cromolyn was found to notably inhibit Myc-induced pancreatic islet tumors,^514^ experimental pancreatic and thyroid cancer,^517–519^ prostate cancer,^520^ and cholangiocarcinoma.^521^ Conclusively, blocking the degranulation of mast cells is considered as a promising approach for reducing inflammation and improving anti-tumor therapy.

Mast cells have been reported to play a pro-tumorigenic role in human bladder cancer through the stimulation of estrogen receptor β (ERβ).^522^ Accordingly, a selective EBβ antagonist inhibited mast cell-promoted tumor growth in a murine model of bladder cancer.^522^ Besides, mast cells were also shown to promote the proliferation of colon cancer in vivo, while injection of the Fcε-PE40 chimeric toxin, known to induce the apoptosis of mast cells, led to retrogradation of colon tumor progression in vivo.^523^

In addition, mast cells have been observed to display significant cyclooxygenase and lipoxygenase activities and to release inflammatory lipid metabolites of AA.^524^ In mice, the major cyclooxygenase products of mast cells are PGD_2_ and PGE_2_, while the primary lipooxygenase products are LTC_4_, LTD_4_, and LTE_3_.^524^ In particular, LTB_4_, a mast cell product of 5-lypoxygenase, is known to be a chemoattractant for mast cell progenitors.^525^ In general, these findings indicated that mast cells and their mediators deserve focused consideration as therapeutic targets in different types of cancer.

#### Activating DCs for uptake and presentation of tumor antigens

DCs are specific APCs that function as messengers between innate and adaptive immune responses.^370^ However, tumor cells have been shown to hijack DCs to promote chronic inflammation and prevent TAA presentation, thus accelerating tumor development.^526,527^ Nowadays, modulating the function of DCs to improve cancer immunotherapy is of particular interest.^383^

The maturation of DCs is essential for providing co-stimulatory signals to T cells. However, although the maturation of DCs occurs within TME, it is often insufficient to induce robust immunity.^384^ Bypassing suppressive pathways or directly activating DCs could trigger a T-cell response, and as such, therapeutic targeting of DCs holds translational potential in combinatorial approaches. The maturation of DCs is important for the initiation of Ag-specific T-cell responses. Nevertheless, the TME also contain a network of immunosuppressive factors (e.g., IL-6, M-CSF, PGE_2_, and VEGF) that could inhibit the infiltration of DCs and subdue their anti-tumor activity.^527^ Therefore, targeting these immunosuppressive pathways might improve the recruitment, infiltration, and anti-tumor activity of DCs in the TME. For instance, VEGF, correlated with poor prognosis in patients with different types of cancer, was found to inhibit the differentiation and maturation of DCs *via* the activation of VEGFR-1 and VEGFR-2.^528,529^ Moreover, blockade of the VEGF signaling significantly increased the proportion of mature DCs in patients with cancer; as a result, the inhibition of VEGF pathways has become an appreciated approach for the treatment of cancer.^530^ In addition, high levels of IL-6, known as a pro-inflammatory cytokine, were associated with a functional defect in DCs from patients with cancer.^531^ The IL-6-induced suppression of DCs could be intercepted by the AG490 JAK2/STAT3 inhibitor,^487,532^ indicating that the pro-inflammatory IL-6/JAK/STAT3 signaling pathway is a promising target for cancer immunotherapy. Moreover, due to the elevated levels of PGE_2_ and activity of COXs in patients with colon cancer and its correlation with tumor size and patient survival, PGE_2_ has been proposed as the principal prostanoid associated with CRC. Furthermore, PGE_2_ was reported to be responsible for the reduced differentiation of DCs from CD34^+^ precursors;^533^ and to mediate DC tolerance *via* the upregulation of the expression of indoleamine 2,3-deoxygenase (IDO1) in DCs, resulting in the differentiation of Treg cells and the inhibition of antigen-specific stimulatory potential of DCs.^525^ In addition, tumor-derived CSF-1 could inhibit the differentiation of hematopoietic progenitor CD34^+^ cells into DCs and induce the differentiation of cord blood monocytes to tolerogenic DCs.^534^ Evidence has shown that the regulation of DCs differentiation driven by CSF-1 was mediated by the PI3K-dependent pathway, delaying the activation of caspases in monocytes.^535^ Hence, removing or blocking immunosuppressive factors (e.g*.*, IL-6, M-CSF, PGE_2_, IL-10, and VEGF) on DCs would promote anti-tumor efficiency by recruiting and promoting the maturation of DCs.

To launch a robust antigen-specific anti-tumor response, some immunostimulatory cytokines targeting the activation of DCs are applied in the development of therapeutic vaccines. First, GM-CSF was demonstrated to serve as a potent immune adjuvant inducing long-lasting anti-tumor immunity.^536^ Growing evidence have suggested that GM-CSF promotes the activation of DCs and enhances TAA presentation to T cells.^537^ In addition, studies revealed that treatment with GM-CSF and IL-4 in vitro could lead to the generation of bone marrow (BM)-derived DCs in mouse and monocyte-derived DCs from human peripheral blood mononuclear cells (PBMC); as a result, these findings have accelerated the clinical applications of GM-CSF.^538^ However, it was shown that in clinical trials administration of a high dose of GM-CSF resulted in immunosuppression, indicating a more complex role of GM-CSF in cancer immunotherapy.^539,540^ Second, the cytokine Fms-related tyrosine kinase 3 ligand (FLT3L), activating signaling through its FLT3 receptor expressed on DC precursors, was observed to be essential during the development of DCs.^541^ Culturing mouse BM precursors with recombinant FLT3L (rFLT3L) was sufficient to generate DCs in vitro.^542^ Furthermore, administration of rFLT3L in mice resulted in significant expansion of conventional DCs and plasmacytoid pre-DCs in vivo,^543^ whereas genetic ablation of FLT3L caused a marked decrease in these subsets.^544,545^

Typically, DCs can recognize a wide range of “danger signals” from both invading microbes and injured host cells through binding either PAMPs or DAMPs to specialized PRRs, such as TLRs, c-type lectin receptors, stimulator of interferon gene (STING), NOD-like receptors, and the RIG-I and MDA5 DNA/RNA receptors.^370^ Accumulating evidence has indicated that activating certain innate immune signaling pathways, especially TLRs, RLRs, and STING signaling pathways, might be a promising cancer immunotherapeutic approach.^546^ For example, the Coley toxin and *Bacillus Calmette-Guerin* TLR2 and TLR4 agonists have become common-used therapeutic agents against several cancers.^547^ The extract of larix leptolepis (ELL) was also found to potentially initiate TLR2 and TLR4 signaling in BM-derived DCs, inducing the activation of tumor-specific CTLs against cancers.^548^ According to these findings, combination therapy using TLR ligands and conventional radiation/chemotherapy, which displays a much higher growth-inhibitory effect compared with single application, might be a promising strategy for cancer treatment.^549,550^ Traditional therapy usually leads to the release of tumor antigens, which are subsequently phagocytosed and presented by macrophages and DCs. Stimulation of TLRs is known to further enhance the maturation of DCs, antigen presentation, and priming of tumor-specific CTLs, resulting in a more effective immunotherapy.^551–553^ Similarly, inducing the activation of melanoma differentiation-associated gene 5 (MDA5) using synthetic double-stranded RNA-poly (I:C) in OVC resulted in the increased expression of HLA-class I, release of cytokines (e.g., CXCL10, IL-6, and type I-IFN), and tumor cell apoptosis.^554^ This MDA5-mediated anti-cancer response was shown to require the DC-dependent phagocytosis of MDA5-activated tumor cells and subsequent generation of cytokines (CXCL10 and IFN-α), providing a pro-inflammatory milieu for boosting the cytolytic function and IFN-γ secretion of NK cells at the tumor site.^555^

Similar to the RLR signaling, activation of the STING pathway results in the production of IFNs for the induction of interferon-stimulated genes (ISGs), prompting cell death in an IFN-independent manner.^556,557^ Moreover, STING signaling has been shown to enhance the expression and secretion of inflammatory cytokines, such as IFNs, TNF-α, and IL-6.^558^ Thus, STING signaling could directly trigger tumor cell death and might provide a new cancer therapy option. Therefore, activation of RLRs could promote endogenous NK or CD8^+^ T-cell-mediated anti-tumor immune responses, and provide a promising approach for anti-cancer immunotherapy.

Radiotherapy is an effective treatment commonly used for various primary and metastatic cancers. In addition to directly destroying cancer cells, radiotherapy has been found to also induce a local inflammation with high levels of IFN-γ and ROS, whereas reduced levels of cytotoxic mediators and DAMPs.^559^ Furthermore, irradiation was demonstrated to trigger long-range effects through the induction of immunogenic cell death relying on the DC priming of CD8^+^ T cells.^560,561^ Moreover, irradiation-induced DNA damage is known to activate PRR, such as the cyclic GMP-AMP synthase cytosolic DNA sensor, which further activates STING, leading to a type I-IFN response by DCs, and contributing to anti-tumor immunity.^562^ In addition, canonical NF-κB signaling was reported to be necessary for the radiation therapy-induced anti-tumor immune responses.^563^ Combination of radiation with anti-PD1 treatment displayed an abscopal effect, with tumor regression in the nonirradiated secondary tumors.^560^ Through the induction of local inflammation and activation of DCs, radiotherapy might provide a nonpharmacological approach to improve the systemic response of immunotherapy.

Immunotherapy using DC-based vaccines could be an approved approach for boosting immune responses through the stimulation of tumor cell-killing T cells and induction of memory T cells for the prevention of cancer recurrence.^564^ These procedures would involve the isolation or in vitro generation and amplification of autologous DCs, followed by their ex vivo manipulation, including the induction of the maturation of DCs and tumor antigen loading, and finally their reinfusion into patients with cancer.^565^ In phase II studies, vaccination with DCs was shown to induce an immunologic response, increasing the number of TILs and providing overall survival benefit for at least a subpopulation of patients.^566^ Furthermore, injection of a DC vaccine comprising antigen-pulsed DCs induced antigen-specific immune responses in vivo, whereas soluble antigen alone failed to trigger immunity.^567^ Vaccination with cDCs loaded with tumor antigens was reported to synergize with the PD-1 blockade in vivo.^568^ Thus, strategies combining the application of DC vaccines with other therapies would be potential to improve anti-cancer efficacy.

#### Targeting tumor-associated macrophages

It is widely known that TAMs are the main infiltrating inflammatory cells in multiple tumors, contributing to an immunosuppressive environment.^569^ Abundant evidences have highlighted the correlation between high numbers of infiltrating TAMs with tumor progression and resistance to therapies.^570^ Therefore, TAMs are attractive targets for cancer therapies aiming to reduce cancer-promoting inflammation and TAM-orchestrated immune suppression.^569^ To date, several main strategies targeting TAMs have been used for the treatment of cancers and inflammatory diseases: reducing the accumulation of TAMs, direct depletion of TAMs, inducing polarization of M1 macrophages, and augmenting macrophage-mediated phagocytosis.

One strategy for targeting TAMs is to inhibit their recruitment or the infiltration of monocytes/macrophages into tumors. Tumor cell-derived CCL2 is known to be critical for the recruitment and infiltration of monocytes and TAMs in several tumor types, including ESCC, CRC, HCC, and breast cancer.^571,572^ Accumulating evidence have suggested that both CCL2 and its CCR2 receptor are implicated in both the inflammation and progression of tumors. Blockade of the CCL2/CCR2 pathway could effectively suppress the accumulation of TAMs in experimental tumor sites, and improve efficacy in combination with chemotherapy. For instance, blockade of the CCL2/CCR2 signaling inhibited the recruitment of inflammatory monocytes, infiltration and M2 polarization of TAMs, suppressed malignant growth and metastasis, reduced postsurgical recurrence, and enhanced survival in a mouse model of HCC.^572^ Similarly, CCR2-targeted therapy with PF-04136309 in combination with FOLFIRINOX displayed a benefit for patients with borderline resectable and locally advanced pancreatic cancer.^573^ In addition, treatment with bindarit, an anti-inflammatory indazolic derivative that can inhibit the synthesis of CCL2, displayed a potential inhibitory function in tumor progression and metastasis in prostate, melanoma, and breast cancers.^574,575^ Importantly, the anti-tumor effects of bindarit were revealed to be related to its ability of selective interference with the infiltration of TAMs and MDSCs. Taken together, these data indicated that the CCL2/CCR2 pathway might be a potential candidate for inhibiting the recruitment of TAMs into the inflammatory and immunosuppressive TME.

CCL5 is an inflammatory chemokine known to promote the migration of macrophages involved in the immune/inflammatory response.^576^ The CCL5/CCR5 axis has been implicated in tumor development or progression of multiple types of cancer (e.g*.*, gastric cancer (GC), breast cancer, glioblastoma multiforme, and CRC), through the recruitment of TAMs and their polarization toward a M2-like phenotype.^577–580^ Maraviroc, a specific CCR5 antagonist, was demonstrated to reduce the infiltration of monocytes/macrophages in breast cancer, GC, glioblastoma, and advanced CRC.^577,581,582^ Therefore, the CCL5-CCR5 axis might be another chemokine pathway with potential for preventing the recruitment of macrophages.

In addition, CSF-1 has also been found to be involved in the differentiation and recruitment of TAMs to tumor milieus *via* the activation of CSF-1R.^583^ High expression of CSF1 and CSF1R have been correlated with poor prognosis in various cancer types, including breast cancer, GC, and OVC.^584–587^ Notably, chemo-radio and hormonal therapies were shown to exhibit the unwanted effect of upregulating the local expression of CSF-1.^588,589^ Emerging data have shown that blocking the CSF-1/CSF-1R signaling prevented the trafficking of TAMs, thereby achieving a meaningful clinical benefit for patients with cancer in clinical trials.^590–592^ For example, emactuzumab, a humanized monoclonal antibody (RG7155) that inhibits the activation of the CSF-1 receptor (CSF-1R), reduced the density of macrophages and increased the ratio of CD8^+^:CD4^+^ T cells in tumors.^592^ Besides, GW2580, a selective CSF-1R inhibitor, has been shown to reduce the infiltration of macrophages and the volume of ascites in a mouse model of ovarian cancer.^589^ Moreover, combining GW2580 with chemotherapy displayed synergistic results.^593^ GS-1101 is a specific inhibitor of the PI3Kp110δ kinase, whose activation is pivotal for the CSF-1-triggered infiltration of TAMs.^594^ Furthermore, GS-1101 inhibited the CSF-1-induced spreading and invasive capacity of TAMs.^594^ These findings suggested that targeting of the CSF-1/CSF-1R signaling could remove TAMs, leading to anti-tumor immune responses.

The selective elimination of TAMs in TME has also been explored for cancer therapy. One attractive strategy for depleting TAMs within the tumor milieu is the induction of their apoptosis. Several compounds (e.g., zoledronate, clodronate, and trabectedin) and bacterial pathogens have been demonstrated to trigger the apoptosis of macrophages. Bisphosphonates, which are primary agents for several bone diseases, have been used for depleting macrophages, as well as TAMs.^595^ Structurally, bisphosphonates are chemically stable derivatives of pyrophosphate, in which the P-O-P bond has been substituted by the P-C-P bond. Liposome-encapsulated bisphosphonates (e.g., zoledronic acid- or clodroenate-loaded liposomes) have been widely applied for the depletion of TAMs in murine tumor models, resulting in markedly reduced tumor growth and metastasis.^596,597^ Furthermore, treatment with bisphosphonates or their combination with other anti-tumor therapies markedly reduced the recurrence and overall mortality of breast and prostate cancer.^595,598^

As the first marine-derived anti-tumor agent approved for the treatment of soft tissue sarcoma and OVC, trabectedin was also found to suppress the accumulation of macrophages in TME and the production of inflammatory mediators from TAMs, such as IL-6, CCL2, CXCL8, angiopoietin 2, and VEGF.^387,599,600^ Moreover, as TRAIL receptors are more highly expressed in monocytes/macrophages than in neutrophils and lymphocytes, trabectedin was shown to specifically activate caspase-8-dependent apoptosis in monocytes/macrophages.^387,601^ In several types of cancer (e.g., breast cancer and sarcoma), trabectedin reduced the density of TAMs, and inhibited tumor growth in vivo.^387,602,603^ Recently, it was shown that the inhibitory effect of trabectedin on the accumulation of TAMs and production of inflammatory mediators from TAMs contributed to its anti-tumor activity, especially, in inflammation-associated cancers.

As a bacterial pathogen, *Shigella flexneri* is known to specifically induce apoptosis in macrophages. To evaluate the effectiveness of *Shigella*-induced depletion of macrophages in achieving tumor regression in vivo, Galmbacher et al. developed an attenuated strain of *S. flexneri*, termed M90TΔaroA.^604^ Interestingly, injection of M90TΔaroA led to caspase-1-dependent apoptosis of TAMs and a significant reduction in tumors of transgenic MMTV-HER-2 mice. Furthermore, depletion of TAMs was associated with complete tumor regression, suggesting that bacterial pathogens might serve as potential arms in the targeting of TAMs for attenuated inflammation and anti-tumor therapy.^604^

The polarization of macrophages has been shown to have a profound impact on inflammation and TME, with repolarization of macrophages from the pro-tumor phenotype (M2) into cytotoxic anti-tumor effectors (M1) improving the TME. Classically, M1 polarization of macrophages can be obtained by stimulation with pathogen-derived LPS alone or in combination with Th1 cytokines, such as IFN-γ and GM-CSF, whereas IL-10, TGF-β, and PGE_2_ contribute toward the M2 phenotype and have been associated with a suppression of inflammation and anti-tumor activities.^605^

Decades ago, the intravesical inoculation of the muramyl tripeptide phosphatidylethanolamine (MTP-PE), a synthetic analog of the muramyl dipeptide (MDP), was applied in the treatment of bladder carcinoma with the aim to activate cytotoxic macrophages against tumor cells.^606^ Besides, stimulation of the TLR signaling by LPS is known to trigger the translocation of NF-κB into the nucleus and the production of inflammatory cytokines, such as IL-1β, IL-6, IL-12, IL-23, and TNF-α.^607^

IFN-γ is a prototypic cytokine inducing the M1 polarization of macrophages, antimicrobial and anti-tumor effects, as well as the enhancement of antigen processing and presentation pathways.^608^ Upon exposure to IFN-γ, macrophages were shown to become primed for induction of the NF-kB pathway through direct and indirect mechanisms.^609,610^ Recently, recombinant IFN-γ displayed antiproliferative, antiangiogenic, and proapoptotic effects on cancer cells,^611,612^ as well as anti-inflammatory effects.^613^ Thus, IFN-γ has been adopted in the clinical management of a variety of malignancies, including bladder carcinoma, CRC, OVC, and adult T-cell leukemia.^614–616^ In general, IFN-γ has been expected to act as sine qua non in the clinical efficacy of anti-tumor immunotherapies.

More recently, an alternative approach has been explored to re-educate macrophages *via* the activation of the CD40 co-stimulatory molecule.^617^ Preclinical experiments have demonstrated that CP-870,893, an agonistic anti-CD40 antibody switches TAMs toward an M1-like phenotype and endowed them with antigen-presenting capabilities, leading to the reestablishment of immune surveillance and retardation in tumor growth.^618^ Moreover, in phase I trials, treatment with CP-870,893 in combination with gemcitabine showed a partial therapeutic benefit in patients with advanced pancreatic cancer.^619^ These findings suggested that the CD40 pathway could be therapeutically harnessed to reverse immune suppression by targeting TAMs involved in cancer-related inflammation.

The STAT3 transcriptional factor is a pivotal regulator of the inflammatory response of macrophages, as well as other immune cells.^620^ Evidence have demonstrated that tumor-secreted lactate induces the M2 polarization of macrophages through the activation of the ERK/STAT3 signaling pathway, thus promoting breast cancer progression.^263^ Likewise, activation of the IL-6/STAT3 pathway was reported to accelerate the progression of HCC *via* switching the M1/M2 polarization of macrophages.^621^ Blocking of the STAT3 signaling was reported to be part of the antiproliferative and anti-inflammatory properties of resveratrol.^622,623^ Furthermore, treatment with metformin suppressed the monocyte-to-macrophage differentiation *via* the AMP-activated protein kinase (AMPK)-mediated dephosphorylation of STAT3.^624^ These data suggested that the anti-inflammatory and anti-tumor properties of the STAT3 signaling inhibitors were related to the M1/M2 polarization of macrophages.

The scavenger receptor macrophage receptor with collagenous structure (MARCO) has been shown to be mainly expressed on macrophages or DCs, regulating inflammatory responses to bacterial pathogens though the recognition of PAMPs.^625^ Mounting evidence has recently indicated that MARCO also plays important roles in regulating the polarization of macrophages.^625–627^ For instance, MARCO is known to be an initial signaling receptor for asbestos, polarizing alveolar macrophages toward a profibrotic M2 phenotype.^626^ Besides, MARCO has been found to be also highly expressed on M2-TAMs with immunosuppressive gene signatures in the TME of both murine tumor models and human cancers. Blocking MARCO using antibodies led to reduced tumor growth and inhibition of metastasis, and a switch to the M1 phenotype of macrophages.^626^ Of note, MARCO could also be used as a viable target for reeducating TAMs.

Apart from depleting TAMs and suppressing the progression of glioma in preclinical murine models, BZL945 was demonstrated to induce TAMs toward a M1 phenotype, unlike other CSF-1R inhibitors.^591^ Administration of polyethylenimine-coated superparamagnetic iron oxide nanoparticles (EI-SPION) could inhibit the M2 polarization of macrophages by suppressing the phosphorylation of Src and cortactin.^628^ Moreover, by downregulating the placental growth factor (PlGF), the host-produced histidine-rich glycoprotein (HRG) skewed TAMs from a M2- to a M1-like phenotype, resulting in reduced tumor growth and metastasis, as well as improving chemotherapy.^629^

Macrophage-mediated phagocytosis is a pivotal process underlying the anti-tumor effect of activated macrophages.^331^ The signal regulatory protein α (SIRPα) expressed on (e.g., macrophages and DCs) was observed to trigger a cascade of events that inhibited phagocytosis when phagocytes interacted with CD47 highly expressed on cancer cells.^630,631^ Thereby CD47 appears to serve as a “don’t eat me” signal and a myeloid-specific immune checkpoint. Currently, antibodies that antagonize the CD47-SIRPα interaction were found to induce the antibody-dependent cellular phagocytosis (ADCP) of tumor cells, and the priming of tumor-specific T-cell responses. Several antibodies targeting the CD47-SIRPα interaction (e.g., Hu5F9-G4, TTI-621, CC-90002, and ALX-148) are being currently tested in clinical trials for both solid and hematologic malignancies.^632,633^ In addition, CD24 expressed in OVC or triple-negative breast cancer was reported to facilitate immune evasion through its interaction with the sialic-acid-binding Ig-like lectin 10 (Siglec-10) inhibitory receptor of TAMs.^634^ Blockade of the CD24-Siglec-10 interaction remarkably strengthened the phagocytosis of all CD24-expressing human tumors,^634^ highlighting that CD24 signaling might be a promising target for cancer immunotherapy.^635^ These findings suggested that targeting the “don’t eat me” signal could promote macrophage-mediated phagocytosis, leading to an anti-tumor innate immune response.

#### Targeting myeloid-derived suppressor cells

MDSCs represent a heterogeneous population of immature myeloid cells with anti-inflammatory effects and potent immunosuppressive activity that play a crucial role in TME.^348^ The accumulation and activation of MDSCs have been shown to correlate with tumor progression, metastasis, and recurrence of many types of tumors.^636^ Moreover, the efficacy of immunotherapy was negatively correlated with an increased density and activity of MDSCs.^637,638^ Hence, targeting MDSCs could become a promising approach to overcome tumor-mediated immunosuppression and enhance the efficiency of tumor immunotherapies. The modulation of MDSCs was achieved by facilitating their differentiation into mature myeloid cells, thus inhibiting their development, expansion, and function, as well as depleting their numbers.

As the immune suppressive phenotype of MDSCs is known to depend on their immature state,^348^ forcing their differentiation into mature myeloid cells (e.g., DCs or macrophages) would impair their suppressive function. It has been demonstrated that agents that can induce the differentiation of MDSCs include vitamin A and D, all-trans retinoic acid (ATRA), IL-12, the activation of TLR9, taxanes, beta-glucan particles, the inhibition of tumor-derived exosomes, and very small size proteoliposomes.^639–641^

Although the mechanism remains unclear, vitamins A and D have been demonstrated to promote the differentiation of MDSCs into mature cells. Compared with vitamin A-replete mice, vitamin A-deficient mice exhibited increased numbers of MDSCs in bone marrow, spleen, and peripheral blood.^642^ Similar results were also observed in tumor-bearing mice and patients with NSCLC.^643,644^ Furthermore, administration of high dose vitamin D reduced the numbers of immature myeloid cells, and increased the levels of IL-12 and IFN-γ, displaying an anti-tumor effect.^645^ ATRA, a vitamin A derivative, was reported to lead to the differentiation of MDSCs to DCs and macrophages by blocking the transduction of the retinoic acid signal in both patients with cancer and tumor-bearing mice.^639,646^ Mechanistically, administration of ATRA led to reduction in the levels of ROS in MDSCs by activating the ERK1/2 pathway.^647^ In addition, in vivo administration of ATRA notably reduced the number of MDSCs, whereas concomitantly boosting CD4^+^ and CD8^+^ T-cell-mediated tumor-specific immune responses. Depletion of MDSCs using ATRA in patients with small-cell lung cancer, dramatically enhanced the efficiency of a DC vaccine against p53 by increasing the levels of CD8^+^ T cells.^648^ In a phase I/II study, ATRA was demonstrated to significantly decrease the immunosuppressive function of MDSCs and increase the levels of CD8^+^ T cells in patients with melanoma receiving ipilimumab therapy compared with patients receiving ipilimumab alone.^649^

Taxanes (e.g., docetaxel and paclitaxel), a class of chemotherapeutic drugs, were also reported to facilitate the differentiation of MDSCs in mice and humans with cancer.^650,651^ Administration of docetaxel reduced the number of MDSCs, decreased their function, whereas increased the activity of CD8^+^ T cells in tumor-bearing mice.^652^ Low-dose paclitaxel promoted the differentiation of MDSCs toward DCs in vitro in a TLR4-independent manner.^653^ In a phase II study, women with HER-2 negative breast cancer treated with doxorubicin and cyclophosphamide followed with docetaxel displayed a complete response and lower levels of circulating MDSCs.^654^

Besides, treatment with β-glucan has been reported to lead to the maturation of M-MDSCs in vitro, and the suppression of the activity of M-MDSCs in tumor-bearing mice, thereby leading to delayed tumor progression.^655^ To date, there have been several ongoing or completed clinical trials evaluating the effects of β-glucans on cancer therapy.^656^ Moreover, tumor-derived exosomes, enriched in proteins and nucleic acids, were observed to prevent immature bone marrow myeloid cells from becoming mature DCs.^657^ Inhibiting the production of exosomes using dimethyl amiloride heightened the anti-tumor efficacy of cyclophosphamide in vivo.^658^ Furthermore, very small size proteoliposomes, such as nanosized particles formed from the outer membrane vesicles of *Neisseria meningitidis* and GM3 ganglioside, were found to induce the maturation of DCs and an anti-tumor response from CD8^+^ T cells in mice.^659^ These studies indicated that inducing the differentiation of MDSCs in patients with cancer might augment immunotherapeutic approaches.

The expansion and infiltration of MDSCs depend on several signaling pathways, including JAK/STAT, VEGF, CXCR2, and COX2/PGE_2_.^660^ First, STAT3 is the main transcription factor regulating the immunosuppressive activity of myeloid cells, and blockage of the STAT3 pathway by various inhibitors can decrease the number of G-MDSCs. For example, the herb derivative curcumin, which is a regulator of STAT3 signaling, was found to exhibit several pharmacologic effects (e.g., anti-inflammatory, antioxidant, and anti-cancer activities), as well as reduce the number of MDSCs at tumor sites and in the blood of tumor-bearing mice.^661,662^ The siRNA-inhibition of STAT3 eliminated the immunosuppressive effects of MDSCs on CD8^+^ T-cell functions by reducing the expression of Arg-1.^663^ However, small molecule inhibitors targeting STAT3 displayed limited efficacy and broad side-effects.^664^

Another strategy to prevent the expansion and infiltration of MDSCs was shown to be the blocking of local signaling in TME, for instance, by targeting VEGF. Although VEGF might promote tumor growth through several mechanisms, including angiogenesis and metastasis, evidence have indicated that the anti-tumor effect of the VEGF blockade was related to its inhibition of MDSCs. In a mouse model of metastatic renal carcinoma, neutralization of VEGF by bevacizumab reduced the CD11b^+^VEGFR1^+^ population of MDSCs in the peripheral blood.^665^ Clinical studies further confirmed that bevacizumab could decrease the number of G-MDSCs, whereas increase the numbers of mature circulating DCs in patients with metastatic CRC, as well as in patients with NSCLC and patients with RCC.^666,667^

The stem cell factor (SCF-1), which is highly expressed in tumor cells, has been shown to enhance the development and expansion of MDSCs.^668^ Decreasing the levels of SCF-1 in mice using tyrosine kinase inhibitors, such as sunitinib, pazopanib, and sorafenib was shown to inhibit the development of MDSCs in the bone marrow.^669^ Blocking CSF-1 by sunitinib agents also decreased the numbers of MDSCs in patients with RCC and RCC-bearing mice.^670^ Furthermore, blocking CSF-1 also increased anti-tumor responses, tumor shrinkage, and prolonged survival in murine models of colon carcinoma and lung cancer.^494,671^ Recently, there exist a number of ongoing clinical trials evaluating the levels of CSF-1 in tyrosine kinase therapy.

Semaphorin 4D (Sema4D) is a proangiogenic cytokine produced by several malignancies, known to induce the expansion of MDSCs from monocytes.^672^ Preclinical studies have demonstrated that antibody neutralization of Sema4D disrupted the expansion of MDSCs, which was associated with a shift in the cellular phenotypes and tumor-derived cytokines toward a pro-inflammatory and anti-tumor milieu.^672^ Treatment with humanized IgG4 mAb targeting Sema4D (VX15/2503) was shown to be well tolerated in patients with advanced solid tumors in a phase I trial.^673^ These results suggested that Sema4D might be a promising therapeutic target for the enhancement of the anti-tumorigenic inflammatory response in HNSCC and other epithelial malignancies.

The CXCR1 and CXCR2 chemokine receptors are known to recruit immune suppressive cells, such as MDSCs into the TME. Blocking the CXCR2 chemokine signaling using anti-CXCR2 mAb was reported to disrupt the CXCR2-mediated tumor trafficking of MDSCs, and improved the therapeutic efficacy of anti-PD1 therapy.^674^ Other approaches inhibiting the infiltration of MDSCs in patients include the inhibition of galectin-3 by GR-MD-02 and the inhibition of CCR2 by CCX872.^675,676^ In general, targeting cytokine or chemokines related to the expansion and infiltration of MDSCs has been shown to significantly improve the efficacy of anti-tumor immunotherapy.

In addition to tumor-derived cytokines or chemokines, the bioactive lipid PGE_2_ has been suggested to drive the expansion and infiltration of MDSCs in patients and experimental murine models.^677^ In response to doxorubicin, PGE_2_ is known to be secreted by tumor cells, increasing the recruitment of MDSCs into the TME.^678^ Moreover, it has been found that in CRC, the receptor-interacting protein kinase 3 (RIPK3) is downregulated in MDSCs in response to PGE_2_, causing an increase in the expression of Arg-1, as well as an NF-κB-mediated upregulation of COX2, thus creating an immunosuppressive positive feedback loop.^679,680^ Celecoxib, a specific COX2 inhibitor, prevented the expansion of MDSC subtypes and reduced the function of MDSCs in a murine model of mesothelioma, improving dendritic cell-based immunotherapy.^681^ In addition, targeting E-type prostanoid receptors (EPs) is another efficient approach for the suppression of the PGE_2_-mediated expansion of MDSCs. Combination of the E7046 EP4 antagonist^682^ with agents targeting Treg cells, was observed to increase the ratio of CD8^+^ CTLs to Tregs and induce the differentiation of MDSCs.^683^ Moreover, the COX2/PGE_2_ signaling has also been found to contribute to the immunosuppressive activity and PD-L1 upregulation of other myeloid cells (e.g., M2-like macrophages). It was further revealed that pharmacological blocking COX2 (celecoxib or aspirin) synergized with ICB therapy in preclinical models.^684,685^ Beyond that, the expansion and infiltration of MDSCs could also be stimulated by many soluble factors in TME, such as granulocyte-macrophage colony-stimulating factor (GM-CSF), G-CSF, M-CSF, TGF-β, TNF-α, S100A9, S100A8, IL-1β, IL-6, and IL-10.

After infiltrating into TME, MDSCs utilize multiple mechanisms including oxidative stress and nutrient depletion to silence the function of effector cells.^686^ The modulation of oxidative stress was shown to be beneficial for controlling the MDSC-mediated immune suppression in tumor-bearing mice. For example, nitroaspirin, generated from the coupling of a NO-releasing moiety to the non-steroidal anti-inflammatory drug aspirin, has been demonstrated to possess properties, such as scavenging of ROS and inhibition of the release of pro-inflammatory cytokines, feedback inhibition of the activity of iNOS, and suppression of cancer cell proliferation.^687^ Oral administration of nitroaspirin restored immune responses in tumor-bearing mice, and increased the number and function of tumor antigen-specific T cells.^688^ In addition, inhibitors of phosphodiesterase-5 (PDE5) attenuated the activity of Arg-1 and iNOS and weakened the suppressive activities of MDSCs in tumor-bearing mice, resulting in suppressed tumor growth with greater infiltration and activation of T cells.^688^ Recently, a trial of tadalafil, another PDE5 inhibitor, displayed positive effects on the activation of CD8^+^ and CD4^+^ T cells, and improved the clinical outcome in patients with metastatic melanoma.^689^

Targeting the critical molecules in the signaling pathways shared by tumors and MDSCs, such as AMPK, HIF-1α, and PI3K, is also being actively investigated. Metformin has been reported to have a broad impact on AMPKα and HIF-1α, which are critical for the expression of CD39 and CD73 in MDSCs.^690^ Moreover, metformin can directly reduce the CD73/CD39-mediated suppressive functions of MDSCs. Importantly, metformin improved the functionality of CD8^+^ T cells and increased the overall survival in patients with OVC.^691^ Pharmacological targeting the highly expressed PI3Kγ on MDSCs using IPI-549 has been shown to be beneficial when in conjunction with the administration of immune checkpoint blocking antibodies.^692^ The efficacy of targeting PI3Kγ in promoting CTLs is currently being evaluated in a phase I clinical trial.^693^ PI3K-δ is an identified critical signaling molecule in Treg cells, and duvelisib, the dual inhibitor of PI3K-δ and PI3K-γ, has been shown to promote the efficacy of immune checkpoint antibodies in a preclinical study.^694^ Duvelisib showed a synergistic anti-tumor effect when combined with treatment with anti-PD-1 and anti-OX40 in an A20 B-cell lymphoma model; this effect was associated with a remarkable reduction in Treg cells, M2 macrophages, and MDSCs.^694^

Chemotherapeutic agents are known to employ a variety of mechanisms to rapidly destroy dividing cells, including MDSCs. In addition to apoptosis of tumor cells, some cytotoxic anti-cancer agents, including paclitaxel, doxorubicin, trabectedin, gemcitabine, docetaxel, and 5-Fu, have been reported to activate the immune system through the depletion of MDSCs.^678,695,696^ Hence, antibodies targeting MDSC-specific markers would serve as an effective approach for the depletion of MDSCs. For instance, anti-Gr-1 mAb enhanced the function of CD8^+^ T cells and delayed tumor progression in tumor-bearing mice, while the efficacy of the anti-Gr-1 mAb in the depletion of MDSCs was potently biased toward young mice.^697^ Moreover, in a clinical trial, DS-8273a, a mAb against the R2 TRAIL-death receptor, was shown to selectively deplete MDSCs without affecting mature myeloid cells and lymphoid cells in patients with advanced solid tumors.^698^ Employing a competitive peptide phage display platform, a new peptide-Fc fusion recombinant protein specifically targeting MDSCs was successfully identified.^699^ Intravenous injection of this peptide completely eliminated both G-MDSCs and M-MDSCs from the blood, spleen, and tumors in vivo, and inhibited tumor xenograft growth compared with controls and treatment using Gr-1 mAb.^699^ Therefore, therapeutic approaches targeting MDSCs would shed new lights on cancer immunotherapy, especially, inflammation-related cancer.

#### Targeting tumor-associated neutrophils

In cancer, neutrophils make up a central component of the immune cells that infiltrate tumor tissues. It has been shown that accumulation of TANs is correlated with the progression and poor outcome of patients with cancer, especially in HCC, ICC, HNC, NSCLC, and RCC.^700^ Similar to TAMs, TANs are also classified into N1 anti-tumor and N2 pro-tumor subsets, with neutrophil polarization influencing the role they play in the TME.^335^ Tumor-associated N2 neutrophils, characterized by high expression of CXCR4, VEGF, and B/MMP9 gelatinase, are known to exert pro-tumoral activities. Neutrophil-targeting agents are being currently developed for the treatment of inflammatory or autoimmune diseases.^701,702^ Growing evidence have suggested that neutrophils might regulate the innate and adaptive immune system during tumor evolution.^339,703^ Therefore, TANs might serve as a promising target for anti-cancer therapies.

The prominent immunosuppressive TGF-β cytokine within the TME has been reported to induce a population of TANs with a pro-tumor N2 phenotype, suppressing the cytotoxicity of neutrophils and restricting their entry into the tumor.^335,704^ TGF-β is abundant both at primary and metastatic tumors, and neutrophil cytotoxicity is not evident in these sites, whereas the pro-tumor functions are manifested. In murine tumor models, blocking the TGF-β signaling using the SM16 TGF-β receptor inhibitor or anti-TGF-β antibodies enhanced the recruitment of cytotoxic N1-neutrophils into tumor sites and activated CD8^+^ T cells, resulting in the recession of tumor growth.^335^ Beyond that, recent studies have demonstrated that inhibition of TGF-β could serve as a promising strategy for the induction of the infiltration of CD8^+^ T cells, improving immunotherapy.^705,706^ In phase I/II trials, administration of GC1008 (fresolimumab, a human anti-TGFβ) showed preliminary evidence of anti-tumor activity and acceptable safety in patients with advanced malignant melanoma, RCC, pleural mesothelioma, as well as breast cancer.^707–709^ Likewise, the combination of galunisertib, a small-molecule selective inhibitor of the TGFβ receptor I, and sorafenib showed acceptable safety and a prolonged overall survival outcome in patients with HCC.^710^ Additional clinical trials have demonstrated that galunisertib enhanced the anti-tumor immunity of other agents, such as gemcitabine, nivolumab (anti-PD1), and durvalumab (anti-PD-L1) in multiple types of metastatic cancers, including HCC, breast, and pancreatic cancer.^711,712^

Type I-IFNs, which were initially identified as having antiviral functions, have been found to possess a N1-promoting effect that opposes that of TGF-β.^713^ Through the activation of various immune cells including neutrophils, T cells, NK cells, DCs, and macrophages, type I-IFNs showed potent anti-tumor function and inflammation regulation activities.^457^ For instance, the deficiency of type I-IFN was shown to lead to a higher metastasis load with a massive accumulation of N2-TANs, which was characterized by the high expression of prometastatic proteins (e.g., S100A8, S100A9, VEGF, and MMP9), in the lungs of a premetastatic murine model.^714,715^ In contrast, compared with untreated patients, the numbers of neutrophils in type I-IFN-treated patients were significantly decreased.^715^ In addition to suppressing the expression of proangiogenic factors, such as VEGF and MMP9, IFN-β enhanced the recruitment of neutrophils and their life span in tumor sites, thereby inhibiting tumor growth.^716^ Thus, enhancing the activity of IFNs at the TME could promote the anti-tumor cytotoxicity of neutrophils and might be considered as an additional strategy in anti-tumor immunotherapy.

G-CSF is a potent growth factor stimulating the biogenesis of neutrophils from progenitors and their trafficking from the BM to blood circulation.^334,717^ More importantly, blockade of G-CSF has been reported to not only lead to a decrease in the numbers of neutrophils, but to also induce a phenotype alteration characterized by the reduced levels of ROS and increased expression of Rb1. Therefore, the tumor-derived G-CSF is responsible for both the development and the activity of TANs in TME, providing a promising target for restricting immunosuppressive TANs. A number of studies have demonstrated the potential beneficial effect of inhibiting G-CSF in improving the anti-cancer therapeutic efficacy. For example, anti-G-CSF treatment could also induce anti-tumor immunity through the activation of NK cells, IL12-producing macrophages, as well as CD4^+^ and CD8^+^ T cells in a CRC mouse model.^708^

The CXCR2 axis is known to enhance the efflux of neutrophils from BM to blood, and the TME, thereby promoting tumor progression and metastasis.^718^ Inhibition of the CXCR2 signaling by knocking-down CXCL3, a critical ligand for CXCR2, or treating mice with SX-682 reduced the recruitment of TANs and enhanced the response to anti-PD1 therapy.^719^ In addition, the CXCL12/CXCR4 axis has also been shown to be a key retention signal for bone marrow neutrophils.^712^ Blockade of CXCR4 signaling using plerixafor suppressed the infiltration of neutrophils into the TME and improved the antiangiogenic therapy in a CRC mouse model.^720^ Increasing studies have shown the potential of the CXCR4 blockade in reducing immunosuppression and facilitating the immunotherapy response.^721–724^

The hepatocyte growth factor receptor (HGF-R) has emerged as an important regulator of neutrophils in tumor immunity and the response to immunotherapy. Importantly, high levels of HGF in blood have been shown to correlate with increased numbers of neutrophils and poor responses to ICB therapy in patients with metastatic melanoma.^725^ The inhibition of immunosuppressive TANs using cabozantinib, an inhibitor targeting HGF-R, VEGFR2, RET, and AXL, sensitized immunotherapy in a syngeneic murine model of CRC.^726,727^ Moreover, cabozantinib could lead to the infiltration of anti-tumor CXCR4^+^ neutrophils *via* the upregulation of neutrophil chemotactic factors, such as CXCL12 and high mobility group box 1 (HMGB1),^728^ suggesting that the HGF-R blockade could convert neutrophils from a tumor-promoting N2 to a tumor-inhibiting N1 phenotype.

#### Activating NK cells

Recent studies have highlighted the fact that patients with low cytotoxic activity of NK cells have a higher incidence of cancer, indicating that NK cells interfere with tumorigenesis.^729^ NK cells show cytotoxicity against diverse tumor cell types, but in the TME, Treg cells, M2 macrophages, and MDSCs can inhibit the activation and anti-tumor activity of NK cells through a series of mechanisms, such as the secretion of immunosuppressive products or interfering with the complex receptor array.^730^ For example, activated platelets can directly inhibit NK cells, while cytokines and metabolites, including TGF-β, adenosine, PGE_2_, IDO, and others, can directly suppress the maturation, proliferation, and function of NK cells.^400,730^ Some clinical responses to activating T-cell cytotoxicity immunotherapy, antibody-based, and tyrosine kinase inhibitor-based immunotherapy were shown to positively correlate with the activation of NK cells.^731,732^ Generally, overcoming the immunosuppressive TME to restore the function of NK cells is a potential therapeutic option for cancer treatment.

NK cells express a broad variety of activating and inhibitory receptors, which can be targeted by antibodies and soluble ligands to enhance the activity of NK cells.^733^ During infection, the activation of NK cells has been shown to be triggered by multiple pro-inflammatory cytokines, such as IL-2, IL-12, IL-15, IL-18, type I-IFN (IFN-α and IFN-β), and IFN-γ. IL-15 plays an essential role in the regulation of the development and activation of NK cells.^734^ In patients with non-Hodgkin’s lymphomas, high concentrations of serum IL-15 following autologous peripheral blood hematopoietic stem cell transplantation (APHSCT) were associated with better survival.^735^ In addition, systemic administration of recombinant IL-15 could stimulate the activity of NK cells. In a phase I clinical trial of patients with metastatic malignancies, administration of recombinant IL-15 induced the proliferation of NK cells and substantially increased their numbers.^736^ In addition, following haploidentical stem cell transplantation, the IL-15-stimulated infusion of NK cells was shown to induce a clinical response in 4 of 6 patients with pediatric solid refractory tumors.^737^ Furthermore, exposure to IL-2 stimulated signaling from activating receptors of NK cells. A phase I clinical trial evaluating rituximab combined with IL-2 against B-cell non-Hodgkin’s lymphoma, revealed that addition of IL-2 to rituximab therapy was safe and resulted in NK cell accumulation and ADCC activity that correlated with the better responses.^738^ Type I IFN could also preactivate NK cells by activating their receptors. Cyclic GMP-AMP, a second-messenger was reported to activate the STING adaptor protein, stimulating the production of IFN-β, and resulting in the priming of NK cells for cytotoxicity.^739^ Furthermore, oncolytic viruses are known to be able to trigger the recruitment of immune cells and induce anti-cancer responses by activating NK cells and T cells, thus selectively killing cancer cells.^740,741^ In preclinical models, localized therapy using the oncolytic Newcastle disease virus (NDV) induced inflammatory immune infiltrates in distant tumors, making them susceptible to ICB immunotherapy through the activation of NK and T cells.^742^

Disruption of immunosuppression is another strategy to elicit NK cells in the TME. The inhibitory factors of solid tumors are known to be composed by a complex composition of immunosuppressive molecules, such as TGF-β, IL-10, IDO, PGE_2_, VEGF, iNOS, and ROS, produced by regulatory immune cells, such as Treg cells, MDSCs, and M2-TAMs, as well as by tumor cells themselves.^730^ These factors generate a chronic inflammatory and immunosuppressive TME, accelerating tumor progression. Besides, the PD-1 and CTLA-4 immune checkpoints, lymphocyte activation gene 3 protein (LAG3), and T-cell immunoglobulin mucin-3 (TIM3) are expressed in some types of NK cells, with their ligands potentially taking part in dampening NK anti-tumor responses; as a result, blockade of interaction with checkpoint inhibitors boosts the activity of NK cells.^743–745^

Killer cell immunoglobulin-like receptors (KIRs) are the most polymorphic among inhibitory receptors that bind the human leukocyte antigen (HLA) class I receptors. As such, blockage of KIRs using an anti-KIR blocking antibody (lirilumab) is currently tested in clinical trials.^746^ It has been reported that inhibition of the TAM tyrosine kinase receptors (e.g., Tyro3, Axl, and Mer) in NK cells can enhance the antimetastatic potential of NK cells in murine models.^747^ In humans, CD96 is known to be mainly expressed in NK cells, CD8^+^, and CD4^+^ T cells. Accordingly, Cd96 (−/−) mice displayed hyperinflammatory responses to LPS and resistance to 3′-methylcholanthrene (MCA)-induced carcinogenesis and lung metastases. Importantly, blockade of Cd96 using an anti-Cd96 antibody or gene knockout improved the function of NK cells in mice.^748^ After treatment with trastuzumab, NK cells were reported to be activated, expressing the co-stimulatory CD137 receptor in the peripheral blood of women with HER2-expressing breast cancer. An agonistic mAb specific for CD137 enhanced the trastuzumab-mediated cytokine secretion of NK cells and NK cell-mediated cytotoxicity.^749,750^ In addition, natural killer group protein (NKG)- 2A is an intracytoplasmic tyrosine-based inhibitory motifs (ITIMs)-bearing receptor expressed on both NK and T cells. Blocking the expression of the NKG2A inhibitory receptor enhanced the anti-tumor immunity mediated by NK and CD8+ T cells.^751^

It should be noted that NK cells often become anergic in TME, due to the chronic stimulation by putative self-ligands, with the stimulation not being mitigated by inhibitory receptors engaged with self-MHC.^752^ Similar to T cells, NK cells can also be reactivated ex vivo to be applied for adoptive cell transfer therapy.^733^ First, NK cells are transduced with cytokine-encoding genes to promote their expansion after infusion. For instance, when injected into immunodeficient mice, NK cells expressing IL-15 were demonstrated to expand well and infiltrate in multiple tissues; this expansion could be further heightened by treatment with IL-2. Second, a chimeric receptor composed of NKG2D, linked to CD3ζ could directly provide activation signals; so adding the DAP10 adaptor molecule to the vector construct could promote the expression of NKG2D and transduce the activation signals.^379^ NK cells endowed with the additional NKG2D-CD3ζ-DAP10 stimulus displayed a notably greater cytotoxic effect against multiple cancer cells both in vitro and in vivo, whereas toxicity toward nontransformed cells remained low.^753^ In addition, expression of anti-CD19 CAR in NK cells specifically and dramatically augmented the NK cell-mediated killing of leukemic cells.^754^ Given the successful anti-cancer therapeutic approaches involving the blockade of T-cell-directed immune checkpoints, these new combination strategies could also affect the anti-tumor functions of NK cells.

#### Targeting eosinophils

Given that TATE is associated with improved prognosis of some cancer types, promotion of the effector function of eosinophils cold serve as a potential strategy against some tumors. Several studies on preclinical murine tumor models have reported the eosinophil-targeting anti-tumor therapy. For example, eosinophils have been frequently observed in patients following immunotherapy with IL-2,^755^ IL-4,^756^ GM-CSF,^757^ or tumor vaccination.^757^ More specifically, a high count of eosinophils in the blood was associated with the responsiveness of patients to immunotherapy, in particular to ICB.^758,759^

Recruitment of eosinophils from the peripheral blood into tumor sites is known to be regulated by various mechanisms, including chemokines (CCR3 and CCR1 ligands), Th2 cell-derived cytokines (IL-5, IL-4), immunomodulators (GM-CSF, IL-4, and IL-2), as well as danger signal molecules (HMGB1 and IL-33).^338,760^ For instance, IL-5, which is known to be the most specific cytokine to eosinophils, was shown to be responsible for their selective expansion and survival.^338^ Besides, chemokine eotaxin-overexpressing HCC cells were observed to activate eosinophil-mediated anti-tumor immunity in the presence of IL-5.^761^ Furthermore, increased tumor-associated eosinophilia were observed in tumors of patients with bladder cancer following treatment with IL-2.^755^ IL-4 exhibited potent anti-tumor activity when present at the tumor site, accompanied by an inflammatory infiltrate comprised predominantly of eosinophils and macrophages; treatment with IL-4 stimulated systemic eosinophilia, as well as increased the levels of serum and urine major basic protein (MBP), which is an eosinophil granule protein.^756^ Moreover, administration of DAMPs or alarmins displayed anti-tumor immunity though the recruitment and activation of eosinophils into tumor sites.^762,763^

The anti-tumor responses of eosinophils are associated with the degranulation of eosinophils.^764^ For instance, IL-2 immunotherapy has been applied to treat both melanoma and RCC. The anti-tumor effect of systemic IL-2 therapy was also reported to correlate with the degranulation of eosinophils, which could have relied on antibody-dependent mechanisms.^765,766^ Consistent with IL-2, the administration of recombinant IL-4 led to the degranulation of eosinophils in a dose-dependent manner in patients with cancer.^767^ In addition, treatment with recombinant IL-25 was shown to induce eosinophilia, which was correlated with tumor suppression.^768^ Taken together, for some types of cancer, chemokines promoting the recruitment or degranulation of eosinophils appear to be a hopeful approach for improving the efficacy of immunotherapy.

### Modulating the inflammation in acquired immunity

Activated adaptive immune cells, including T and B lymphocytes, are known to further amplify the initial inflammatory response.^769^ More specifically, Th1 cells activate macrophages both through cell-to-cell contact and secretion of IFN-γ,^770^ while Th2 cells activate eosinophils through the release of cytokines, and B cells secrete antibodies that activate the complement cascade, as well as phagocytes, NK cells, and mast cells through Fc receptors.^351,771,772^ However, certain adaptive immune cells, especially Treg cells and Breg cells have been found to be able to turn off the inflammatory response.^773,774^ Thus, activating acquired immunity is a promising new target for cancer immunotherapy and inflammation control.

#### Activating cytotoxic T cells

During the inflammation process, activated CD8^+^ T cells produce IFN-γ, TNF-α, and granzymes, which destroy target cells.^775^ Clinical evidence have shown that the number of TILs, particularly CD8^+^ T cells, is a positive prognostic marker of multiple solid tumors.^776^ However, the effector functions of CD8^+^ T cells have been shown to be gradually lost in TME during chronic inflammation, a condition named T-cell exhaustion.^418^ T-cell exhaustion, in which reduced and dysfunctional effector T cells lead to immune escape, is one of the mechanisms employed by pathogens or tumor cells to get rid of the control of immunologic surveillance.^418^ Currently, exogenous reactivation, or priming of CTLs has been demonstrated to overcome the chronic inflammatory TME, leading to successful immunotherapy strategies against cancer.

It is well known that cGAS–STING-mediated DNA sensing in cancer cells or phagocytes (e.g., DCs, macrophages) is crucial for detecting cytosolic DNA, inducing a type I-IFN response for augmenting anti-tumor immunity, as well as host defense against pathogens.^777^ It has been reported that once activated by ligands, STING aggregates in a perinuclear region and activates the TBK1 kinase, which in turn phosphorylates IRF3, directly launching the transcription of type I IFN genes. Evidence has shown that the STING agonist DMXAA induces a cooperation between T lymphocytes and myeloid cells, resulting in tumor regression in vivo.^778^

Besides, co-stimulatory and co-inhibitory receptors play a crucial role in T-cell biology, as they are known to determine the functional outcome of TCR signaling. Accumulative evidence have suggested that CD40 plays an intrinsic role in the co-stimulation of T cells.^779^ CD40 has been shown to activate multiple signaling pathways, including Ras, PI3K, and protein kinase C, resulting in NF-κB-dependent induction of cytotoxic mediators (e.g., granzyme and perforin), and boosting of CD8^+^ T cells.^780^ CD28, another major co-stimulatory molecule for the priming of T cells, was reported to recruit adaptors, such as PI3K, growth factor receptor-bound protein 2 (GRB2), and LCK, resulting in the activation of nuclear factor of activated T cells (NFAT), activator protein (AP)-1, and NF-κB.^781^ The CD28 signaling was further shown to amplify TCR signaling, including the expression of IL-2 and B-cell lymphoma (Bcl)-2, modulation of metabolism, and epigenetic changes.^781,782^ Nowadays, a substantial amount of drugs targeting co-stimulatory molecules are in clinical trials against cancer, including members of the TNF receptor superfamily, OX40 (CD134), CD27, and 4-1BB (CD137).^783^

One of the primary characteristics of exhaustion is the co-expression of high levels of a series of inhibitory receptors, including PD-1, CTLA-4, CD152, LAG-3, Tim-3, CD244/2B4, CD160, and TIGIT. Importantly, ICB has been found to be able to induce durable responses among multiple types of cancer both in patients and murine model.^784^ Two inhibitory molecules, the cytotoxic CTLA-4 and PD-1, have attracted much attention, because blockage of the CTLA-4 or PD-1 signaling has prominently improved the survival of patients with metastatic solid cancers.^785^ Furthermore, ICB alone or combinations with other immunotherapies, such as adoptive cell therapy and DC vaccination, has displayed some survival benefit for patients with advanced cancer.^785^

In the perspective of anti-tumor immunotherapy, modulating the actions of cytokines is an attractive strategy to control exhausted CD8^+^ T cells. For example, administration of IL-2 has been approved by the U.S. Food and Drug Administration (FDA) for the treatment of metastatic RCC and melanoma.^786^ Moreover, establishing the effective combination of cytokine-targeted therapy and ICB is of great interest. Administration of IL-2 during chronic viral infection was demonstrated to exhibit striking synergistic effects with PD-1 blockade, thus enhancing virus-specific CD8^+^ T cells.^787^ The synergy of exogenous IL-2, PD-1 blockade, and a powerful T-cell vaccine combination therapy has been confirmed in a cancer model.^788^ Besides, blockade of IL-10R significantly improved the efficacy of anti-PD-L1 treatment, resulting in enhancing the function and viral clearance of T cells.^789^

Adoptive T-cell therapy includes the use of gene-modified T cells expressing novel TCR or CAR receptors that recognize tumor cells and carry out potent anti-tumor functions.^790^ CAR-T-cell therapy has shown tremendous success in B-cell malignancies, leading to the approval of this approach for certain types of leukemia and lymphomas by FDA.^791^ For instance, CD19-specific CAR-T cells (Kymriah and Yescarta) have yielded remarkable clinical trial results in the treatment of certain types of B-cell leukemia and lymphomas.^792–794^ However, the efficacy of CAR-T cells in solid tumors is limited to date, partly due to the lack of trafficking of CAR-T cells to the tumor site, insufficient activation of the transferred T cells, and the immunosuppressive TME in solid tumors.^795^

However, CAR-T-cell therapy can trigger a severe inflammatory storm, known as inflammatory cytokine release syndrome (CRS), which can lead directly to death. On a troubling note, the better therapeutic effects of CAR-T cells have been associated with a stronger CRS.^29,30^ T cells kill tumor cells partially by releasing perforin and granzymes. Nowadays, researchers have found that CAR-T cells attack leukemia cells, leading to the swelling and bursting of tumor cells. Subsequently, the lysis of tumor cells has been shown to activate macrophages, which release a large amount of inflammatory factors (e.g., IL-6 and IL-1), inducing CRS. Meanwhile, NK-T cells cause the apoptosis of tumor cells, which are more pyknotic.^796^ In order to prevent the CRS caused by CAR-T therapy, several approaches can be adopted. For instance: (1) shifting the module of death of tumor cells from pyroptosis to apoptosis, thereby controlling the occurrence of CRS; (2) eliminating the ATP-released pyroptosis of tumor cells; (3) suppressing the activation of macrophages. Collectively, restoring the function of exhausted CD8^+^ T cells is a potential strategy for achieving improved therapeutic benefits of anti-tumor immunotherapy.

#### Targeting Treg cells

Treg cells are usually identified as a specialized subset of CD4^+^ T cells functioning in the establishment and maintenance of immunosuppression, such as promoting the resolution of inflammation, suppressing aberrant immune responses against self-antigens, and limiting anti-tumor immune responses. Treg cells are known to suppress pro-inflammatory responses through the secretion of cytokines, such as IL-2, IL-10, IL-35, and TGF-β.^797^ Besides, Treg cells are able to regulate not only T cells but also B cells, NK cells, DCs, and macrophages *via* humoral and cell–cell contact mechanisms. For example, Treg cells have been reported to facilitate the conversion of DCs to a tolerogenic state through the expression of cytotoxic CTLA-4, and inhibit the proliferation of effector T cells (Teff) cells through the production of inhibitory molecules, such as tryptophan and adenosine, reducing the IL-2-dependent activation of CD8^+^ T cells and NK cells.^797^ Clinically, accumulation of infiltrating Treg cells in tumor has often been associated with poor prognosis of patients with cancer.^798^ Accumulating evidence suggesting that depletion of Treg cells or modulation of the function of Treg cells was able to evoke and enhance anti-tumor immune responses. Various molecules relatively specific to Treg cells are good candidates for the depletion or functional modulation of Tregs, such as immune checkpoint molecules (e.g., CTLA-4, GITR, LAG3, and PD-1), CCR4, and metabolites (e.g., PGE_2_, tryptophan, and adenosine) that have been targeted by Abs or small molecules.

As immune checkpoint molecules are known to be highly expressed in Treg cells, they could thus be targeted to control the function of Treg cells. One of the recent breakthroughs in cancer immunotherapy was the clinical use of anti-CTLA-4 antibodies (ipilimumab and tremelimumab), which were shown to induce tumor regression and improve the survival of patients with metastatic melanoma.^799–807^ Although targeting CTLA-4 was initially designed to reactivate Teff cells, CTLA-4 is also highly expressed on Treg cells, with anti-CTLA-4 antibodies inducing the depletion of Treg cells in the TME through the activation of ADCC.^806,807^ In addition, anti-CTLA-4 antibodies have been reported to exhibit complimentary activity with therapies targeting anti-PD-1 (nivolumab), another checkpoint inhibitor expressed on Treg cells, with their combined use being more beneficial than the use of either antibody alone.^808–810^ Furthermore, other molecules expressed by Treg cells with immunosuppressive activity, such as TIGIT, LAG3, and TIM3, are being currently considered and tested in clinical trials.^811–813^ Importantly, targeting TIM3 might be more advantageous than CTLA-4 and PD-1 blockage, because the expression of TIM3 is restricted to intratumoral T cells, and hence its inhibition is less likely to be associated with adverse autoimmune-like toxicities.^814–816^ Indeed, TIM3-deficient mice did not display autoimmune disorders.^817^

GITR, a member of TNFRs providing co-stimulatory signaling to activate T cells, is known to be highly expressed in intratumoral Treg cells. An agonistic antibody targeting GITR was shown to attenuate the activity of Treg cells, reduce their numbers, decrease their stability in tumors, and promote tumor regression in mice,^818^ particularly when combined with treatment with CTLA-4 or PD-1 inhibitors.^819–821^ Similar to CTLA-4 or PD-1 blockade, GITR ligation also improved the functions of Teff cells.^822^ In addition, OX40, another TNF receptor family member, showed similar patterns of expression and function to GITR. Treatment with anti-OX40 mAb impaired the function of Treg cells and enhanced Teff cell responses, resulting in increased anti-tumor immunity and improved tumor-free survival.^823^ Thus, TNFR2 is a potential target for the development of Treg cell-based immunomodulatory therapies.

Low-dose cyclophosphamide has been shown to deplete Treg cells by inhibiting their proliferation and inducing apoptosis, and to attenuate their function by suppressing the expression of FOXP3 and GITR.^824^ In addition, TKIs (sunitinib, sorafenib, and imatinib) have been reported to prevent the expansion and function of intratumoral Treg cells.^825–827^ Although these approaches have been shown to inhibit the proliferation and function of Treg cells, they are not specific to tumor-infiltrating Treg cells. Moreover, antibodies targeting CD25 (e.g., daclizumab, basiliximab, and LMB-2), have been applied for the depletion of Treg cells by inducing ADCC and complement-mediated cytotoxicity.^797^ Preclinical and clinical studies using a combination of anti-CD25 antibodies (DAB389IL-2) and DC vaccines displayed beneficial effects in patients with RCC.^828^ Furthermore, it was reported that in the treatment of relapses of leukaemia in patients who did not develop graft-versus-host disease (GVHD) during the first transplantation, depletion of Treg cells from hematopoietic stem cell transplantation (HSCT) showed better outcomes.^829^ However, global depletion of Treg cells has been shown to have variable efficacy and could potentially induce systemic complications.

Indoleamine 2,3-dioxygenase (IDO), an enzyme with 2 isoforms (IDO1 and IDO2), converts tryptophan to kynurenine, resulting in the exhaustion of tryptophan, a molecule that is crucial to the proliferation and differentiation of T cells. Accordingly, lack of tryptophan and overproduction of kynurenine has been shown to not only diminish the proliferation and survival of T cells, but also shift the differentiation of T cells into Treg cells.^830^ Interestingly, evidence have shown that combined treatment using an IDO inhibitor and a tumor vaccine induce the upregulation of IL-6 in pDCs and in situ, resulting in the conversion of a majority of Tregs to Th17-like cells, with marked enhancement in the activation and anti-tumor efficacy of Teff cells. These findings suggested that the combined application of IDO inhibitors and tumor vaccines could be an alternative strategy for the reduction of Treg cells by converting them to Teff-like cells.^831^

CCR4 is predominantly expressed by effector Treg cells, rather than naive Treg and Th2 cells,^832^ with the migration and infiltration of Treg cells into solid tumors appearing to be dependent on CCR4 ligands (e.g., CCL17 and CCL22).^833,834^ Blocking the interactions between CCL22 and CCR4 by anti-CCL22 antibody reduced the accumulation of intratumoral Treg cells and suppressed tumor growth in mice.^834^ Clinical trials conducted using a humanized anti-CCR4 antibody (mogamulizumab, KW-0761) displayed a depletion of intratumoral Treg cells and anti-tumor activity with minimal or moderate toxicity.^832,835^ Similarly, in a mouse model with orthotopically implanted human OVC cells overexpressing CCL28 (the ligand for CCR10), intraperitoneal injection of an anti-CCR10 immunotoxin led to complete depletion of intratumoral Treg cells, reducing tumor growth.^836^

The effects of anti-inflammatory cytokines (e.g., TGF-β, IL-10, and IL-35) secreted by Treg cells in the TME can be blocked by using neutralizing antibodies. For instance, blockade of TGF-β expressed by Treg cells improved the anti-tumor immune response against melanoma, and suppressed the metastasis of pancreatic tumors in mice.^837,838^ Treatment with anti-IL-35 revealed the ability of suppressing tumor growth in multiple murine models of cancer.^839^ Therefore, depletion or dysfunction of intratumoral Treg cells, together with the augmentation of the tumor-killing activity of Teff cells, would make cancer immunotherapy more effective with less adverse effects.

#### Activating Th1 cells

The malfunction of Th1 cells has been observed in the peripheral blood of multiple types of cancer, involving them in tumorigenesis and tumor progression.^840^ Furthermore, a high density of tumor-infiltrating Th1 cells is considered to be a beneficial prognostic marker in several types of solid cancers, including OVC, CRC, NSCLC, and breast cancer.^841,842^ Clinical trials have demonstrated that inflammation driven by tumor-specific Th1 cells prevented the progression of malignancies,^843^ providing a strong rationale to develop anti-tumor Th1 immunity-activating immunotherapy.

An efficient strategy to stimulate the Th1 response is using a cancer vaccine. In phase I/II trials of patients with NSCLC, a human telomerase reverse transcriptase (hTERT)-derived helper peptide vaccine (GV1001) induced CD4^+^ T cells displaying a Th1 cytokine profile, and stimulated T-cell responses in >50% of subjects, without exhibiting clinically important toxicity.^844,845^ These results indicated a positive correlation between GV1001-specific Th1 responses and prolonged survival. Recently, universal cancer peptides (UCP), novel anti-tumor Th1-inducer peptides derived from hTERT, were demonstrated to induce a spontaneous CD4 T-cell response in 38% of patients with metastatic NSCLC, with the high-avidity UCP-specific CD4 T cells being Th1 polarized.^846^ In addition, tumor cell loaded type-1 polarized DCs induced the activation of antigen-specific Th1-type CD4^+^ T cells, resulting in a significant reduction in tumor growth.^847^

Application of Th1 cytokines has been suggested to reinforce the anti-tumor effects of immunotherapy. Th1-type cytokines, including IL-1, IL-2, IL-12, and GM-CSF are known potent stimulators of the differentiation of Th1 cells and Th1-based anti-tumor response.^848^ Although many preclinical studies have demonstrated the anti-tumor effects of Th1 cytokines, their clinical efficacy remains limited. To date, most studies on cytokine immunotherapy have focused on the mechanism by which to augment the Th1 response. To this end, many studies have combined cytokine-based therapy with other therapies to reverse immunosuppression in TME.^849^ For instance, IL-12 was found to promote the differentiation of Th1 cells and activation of NK cells, as well as increase the production of IFN-γ. More specifically, IL-12 induced Th1 responses against cancers and improved the anti-tumor efficacy of cancer vaccines, DC vaccines, and other cytokines (IL-18 and IL-15).^849^ Besides, IL-12 gene-modified DCs showed positive clinical response in patients with stage IV melanoma.^850^

Many immune adjuvants are known to display potent capability toward enhancing the production of Th1 cytokines and amplifying Th-immunity in response to cancer vaccines. Currently, several important Th1 adjuvants have been used for the activation of Th1 immunity, such as *Bacillus Calmette-Guérin* (BCG), heat-shock proteins (HSPs), TLR9 agonists, and unmethylated cytosine phophateguanosine oligodeoxynucleotides (CpG-ODN).^848^

Evidence has shown that PGE_2_ shifts the balance away from Th1 responses toward Th2 responses.^851^ Overproduction of PGE_2_ has been observed in multiple Th2-associated diseases, including atopic dermatitis and asthma.^852^ Moreover, inhibition of prostaglandin synthesis using COX2 inhibitors was reported to cause an augmentation of the Th1 response,^853^ suggesting that reducing the production of PGE_2_ would induce the Th1 response, improving the efficacy of anti-tumor immunotherapy. Therefore, developing strategies focusing on the activation of the Th1 immunity response would contribute to successful anti-tumor immunotherapy.

#### Targeting Th2 cells

Th2 cells are regulated by the innate immune system through the use of IL-25, IL-33, and TSLP cytokines. Preclinical breast cancer models have demonstrated their important role in cancer development and metastasis. IL-33, an important member of the IL-1 family was shown to play a pivotal role in regulating immune responses through interactions with its suppression of tumorigenicity 2 (ST2) receptor.^854^ Once activated, the IL-33/ST2 pathway has been demonstrated to regulate Th2 immune responses in autoimmune and inflammatory conditions.^855^ In addition, IL-25, another Th2 promoting cytokine, was found to be highly expressed in human and murine breast cancer.^856^ Blockade of IL-25 by an antagonistic antibody decreased Th2 and M2 macrophages in the primary TME and inhibited tumor metastasis.^856^ Thus, restraining the activation of Th2 cells or reducing the production of type 2 effector cytokines would benefit cancer immunotherapy.

#### Targeting Th17 cells

The IL-17-secreting CD4+ T cells have been defined as Th17 cells, and constitute ~1% of CD4+ T cells in the peripheral blood of healthy donors.^857^ Through the secretion of IL-17, IL-17F, and IL-22, Th17 cells play key roles in many human diseases including inflammation, autoimmune diseases, and cancer. Importantly, Th17 cells have been reported in many types of human cancers, impacting the prognosis of patients. For example, high levels of Th17 cells have been associated with improved prognosis of patients with OSCCs,^11^ and salivary gland tumors.^858^ Patients with melanoma, early-stage OVC, and malignant pleural effusions exhibiting increased numbers of Th17 cells were reported to have better survival.^859^ As experimental and clinical studies have demonstrated that targeting IL-17 has achieved great efficacy in autoimmune diseases, such as psoriasis,^860^ it is predictable that manipulation of Th17-cell biology would be a promising therapeutic modality for the treatment of Th17-affected cancers.

In CRC, however, high levels of IL-17 were observed to refrain Th1-armed anti-tumor immunity, in part by attracting myeloid cells into tumors. Deletion or blockade of IL-17 suppressed the tumor-promoting inflammation, reactivated tumor immunosurveillance, and reduced the frequency of tumorigenesis in lung cancer models.^460,861^ Accordingly, patients with CRC could be benefited by cancer immunotherapy using the anti-IL-17 approach as adjuvant therapies, which would contribute to the inhibition of both IL-17-mediated tumor promotion and T-cell exclusion.

Furthermore, the development and function of Th17 cells have been shown to be regulated by innate system-derived pro-inflammatory cytokines, such as IL-6, IL-23, and IL-1. In particular, IL-23 promotes inflammatory responses, such as the upregulation of the MMP9 matrix metalloprotease, and increases angiogenesis, but reduces the infiltration of CD8 T cells.^862,863^ As a result, transplanted tumors were observed to be growth-restricted in IL-23R-deficient mice.^864^ Besides, IL-6 has been shown to stimulate the production of Th2 type cytokines and upregulate the expression of VEGF and NRP-1 in pancreatic cancer cells.^865^ Agents targeting IL-6, IL-6 receptor, or JAKs have already received U.S. FDA approval for the treatment of inflammatory conditions or myeloproliferative neoplasms. Moreover, to reduce the adverse effects of CAR-T cells, combinations with anti-IL-6/IL-6R signaling strategies are being currently evaluated in patients with hematopoietic malignancies and solid tumors.^487,866^

#### Activating B cells

As the central compose of humoral immunity, B-lymphocytes are known to function in the production of antibodies, presentation of antigens, and secretion of inflammatory cytokines.^867^ TIBs can be detected in various solid tumors.^868,869^ Evidence have suggested that in some cancers TIBs inhibit tumor progression by secreting antibodies and cytokines, promoting T-cell response, and directly destroying tumor cells.^427,870^ Nowadays, several strategies have been developed to fully unleash the anti-tumor potential of B cells.

In order to activate cytotoxic T cells against tumors, B-cell-based cancer vaccines have been designed for the stimulation of B cells. In this context, the use of CD40 stimulation has been widely studied. The ligation of CD40 with CD40L was reported to stimulate the expression of co-stimulatory molecules and cytokines, with CD40-activated B cells increasing the potential to facilitate the activation of naive and memory T cells.^871,872^ In addition, it has been shown that these CD40-activated B cells were resistant to the immunosuppressive TME,^873^ and could reach secondary lymphoid organs after being injected in vivo, where they could efficiently activate T cells.^874^ The CD40-activated B cells have been tested and validated in preclinical models of human papillomavirus 16 (HPV16) E6- and E7-expressing TC-1 tumors,^875^ B16-F10 melanoma, E.G7 lymphoma,^872^ 4T1 breast tumor metastasis,^876^ and spontaneous non-Hodgkin’s lymphoma.^871^

CpG-ODN, a TLR9 ligand, can also be used to activate B cells. In a mouse model of B16-F10-derived lung metastases, injection of CpG-activated B cells was reported to cause a regression of metastases and a less immunosuppressive TME.^877^ The GIFT4, a fusion between GM-CSF and IL-4 cytokines, which were found to unexpectedly cluster the respective receptors on B cells, resulted in the activation of the JAK/STAT pathway in B cells.^878^ This clustering could promote the proliferation of B cells and their differentiation from naive B cells toward activate helper B cells. Subsequently, these activated B cells were observed to act as APCs, secreting cytokines and expressing co-stimulatory markers, resulting in the activation of T cells. Administration of GIFT4 to melanoma-bearing mice caused an efficient regression of tumors. Furthermore, tumors were found to be resistant to GIFT4 in B-cell-deficient mice, suggesting that the anti-tumor effect of GIFT4 was B-cell-dependent.^878^

In addition, tumor-derived autophagosomes enriched in defective ribosomal products (DRibbles) were shown to be captured and internalized by B cells in vivo.^879^ These DRibbles contain tumor-specific antigens and activate B cells with increased expression of MHC class I and II molecules, CD86, and CD40. Then, these activated B cells can present DRibbles-derived antigens to stimulate the anti-tumor T-cell response. In lymphoma- or HCC-bearing mice, the combined injection of DRibbles and DRibble-loaded B cells led to the control of tumor growth.^879,880^

#### Targeting Breg cells

In human cancer, the frequencies of regulatory B (Breg) cells have been shown to usually increase with tumor progression, and to be enriched in tumors compared with peripheral blood or adjacent normal tissues. Breg cells are known to mediate inflammation and maintain homeostasis mainly *via* the secretion of anti-inflammatory cytokines, such as IL-10, TGF-β, and IL-35,^881^ directly killing effector cells by expressing Fas-L.^882^ Through the secretion of TGF-β, Breg cells were reported to promote the transformation of effector CD4^+^ T cells to active Tregs, which in turn suppressed the proliferation of T cells and facilitated tumor metastasis.^883^ Moreover, IL-21 was shown to induce granzyme B (GrB)^+^ Breg cells contributing to tumor escape from an efficient anti-tumor immune response.^884^ In addition, Breg cells were observed to dampen immune responses through the inhibition of the differentiation of DCs and the proliferation of Th1 and Th17 cells.^885^ These results suggested that Breg cells play an important role in regulating inflammation and the immunosuppressive TME, which prevent the anti-tumor immune process.

LXA_4_, an endogenous eicosanoid derived from AA, has been highlighted in the regulation of inflammation.^886^ Evidence have shown that LXA_4_ repressed the generation of Breg cells by dephosphorylating both STAT3 and ERK, resulting in impaired tumor growth.^26^ Targeting Breg cells by LXA_4_ decreased the number of Treg cells in tumor tissues, as well as enhanced the activities of cytotoxic T cells.^26^ These findings revealed that targeting Breg cells through the administration of LXA_4_ could have potential clinical applications. Interestingly, another metabolite from this pathway was observed to display the opposing effect: LTB_4_ could trigger the conversion of naive B cells into Breg cells. Furthermore, MK886, a 5-lipoxygenase activating protein (FLAP) inhibitor and antagonist of PPARα, could inhibit the generation of Breg cells, suggesting the crucial role of the 5-LOX/FLAP pathway in the differentiation and suppressive function of Breg cells.^887^

The phytoalexin resveratrol, which is known to block the phosphorylation of STAT3, was demonstrated to cause a decreased proportion of Breg cells in 4T1-bearing mice, inhibiting the formation of lung metastases.^887^ Likewise, total glucosides of paeony (TGP) extracted from plant were found to exert anti-inflammatory and immunomodulatory activities. In a rat model of diethylnitrosamine (DEN)-induced HCC, treatment with TGP resulted in a reduction of nodules and improvement of survival through a reduction in the numbers of Breg cells.^888^

Furthermore, tumor-infiltrating Breg cells with increased expression of PD-L1 and TGF-β suppressed the proliferation of CD4^+^ T cells, CD8^+^ T cells, and NK cells. Monoclonal antibodies targeting TGF-β or PD-L1 notably suppressed tumor growth and reduced the number of Breg cells in mice.^887^ Treatment with ibrutinib was shown to improve the immunosuppressive TME of chronic lymphocytic leukemia (CLL) *via* the STAT3-mediated suppression of Breg cells and the PD-1/PD-L1 pathway.^889^ Taken together, selectively targeting Breg cells in the TME appears to be a hopeful strategy for tumor immunotherapy.

Nowadays, numerous trials designed to evaluate the efficacy of inflammation modulators in cancer prevention and treatment are ongoing. Some ongoing or completed clinical trials with agents targeting cancer-associated inflammation signaling pathways are listed in Table 1. On the one hand, monotherapy with anti-inflammatory agents has been proved limited efficacy in cancer treatment, for example, monotherapy with agents targeting IL-6 only showed moderate activity against solid tumors in non-stratified patients (NCT00841191, NCT01531998). On the other hand, modulating inflammation can boost anti-cancer efficacy in synergy with chemotherapy or immunotherapies, for example, compared with placebo, adjuvant therapy with the celecoxib can benefit disease free survival (DFS) of two years and overall survival (OS) in primary breast cancer patients received endocrine treatment according to local practice (NCT02429427).

Hence, based on the relationship between inflammation and cancer, targeting inflammatory cells or inflammatory factors would contribute to the achievement of better outcomes for cancer therapeutics. The acute inflammation induced by some therapies (e.g., recombination IFN, TLRs activator, STING activator) can redirect the pro-tumor TME toward an anti-tumor immune milieu, which can enhance efficiency of anti-cancer therapies (e.g., chemotherapy, radiotherapy and immunotherapy). Moreover, a number of therapeutic strategies to limit chronic inflammation (incuding systermic and local inflammation) have been successfully applied in clinical or preclinical tumor models to prevent tumorigenesis, or sensitize tumor to anti-cancer therapies including chemotherapy, radiotherapy, and immunotherapy.

## Conclusion and remarks

Acute inflammation is the initial response to harmful stimuli, with the persistence of inflammatory factors potentially inducing chronic inflammation.^46^ Innate immune cells (endothelial cells, neutrophils, macrophages, mast cells, NK cells, and DCs) and adaptive immune cells (T cells and B cells), as well as pro-inflammatory factors (vasoactive amines, vasoactive peptides, complement fragments, and some cytokines, such as IL-1, IL-6, IL-15, IL-17, IL-23, TNF-α, and IFN-γ) are important for the initiation of inflammation. Besides, chemokines (CCL2, CXCL12) are necessary for the recruitment of inflammatory cells in the inflammatory area. However, anti-inflammatory cells (M2 macrophages, Th2, Tregs, and MDSCs), some cytokines (IL-4, IL-10, IL-13, and TGF-β) and SPM (LTA_4_, LXA_4_, LXB_4_, lipoxins, RvE, RvD, MaR_1_, MaR_2_, DHPA, PCTR1, and protectin D_1_) are involved in the resolution of inflammation. The effect of inflammation on most cancers is two-edged, with cancer also affecting the process of inflammation. Normally, the immune system recognizes and removes the pathogens and tumor cells, thus inhibiting tumor growth.^369^ However, during chronic inflammation, inflammatory cells and cytokines might act as tumor promoters affecting cell survival, proliferation, invasion, and angiogenesis.^276^

Based on the close relationship between inflammation and tumor, targeting inflammation is an important way for improving anti-cancer treatment. There are two aspects in targeting inflammation for cancer treatment. Activating anti-cancer immunity cells (e.g., DCs, NK cells, NKT cells, CTLs, Th1 cells, and B cells) can improve the cancer-killing ability of the immune system. Concomitantly, inhibiting procancer immune cells (e.g., mast cells, TAMs, MDSCs, TANs, eosinophils, Th2 cells, Th17 cells, Treg cells, and Breg cells) or converting their polarization to anti-tumor type by targeting key signal pathways can impede the immunosuppressive effect and the progression of cancer. For example, ablating unfolded protein response mediator PERK in MDSC can reverse their pro-tumor role and elicit anti-tumor T cells.^890^ Besides, the intestinal microbiome also played an important role in inflammation and cancer, especially between IBD and CRC.^271,272^ Microbiota are known to directly or indirectly (*via* their metabolites, such as polysaccharide β-dextran, LPS, deoxycholic acid (DCA), short-chain fatty acid (SCFA), butyrate, and propionate) affect the differentiation and function of immune cells (e.g., M2-TAMs, TANs, Treg cells, DCs, and CD8^+^IFN-γ^+^ T cells), potentially altering their effects on tumors.^275–277,279,280,891,892^ Thus, intestinal microbiota are promising targets for the treatment of inflammation-associated cancer and fecal microbiota transplant (FMT) is becoming an effective method to improve the intestinal microbiome.^893^

Although the inhibition of inflammation targeting the innate and adaptive immunity has offered remarkable achievements in the clinical field of cancer therapy, several obstacles and challenges remain exist. Cancer therapy-induced inflammation often endows residual cancer cells with resistance to subsequent courses of treatment, enhancing cancer progression.^894^ For example, the Fc side of the checkpoint antibody could cause ADCC and CDC.^895^ Besides, ipilimumab (anti-CTLA-4) was shown to promote colitis and hypophysitis,^896,897^ while anti-PD-1 therapy improved the incidence of thyroiditis, pneumonia, and diabetes.^897–899^ Moreover, CAR-T therapy could cause a “cytokine storm”, with insufficient persistence of CAR-T cells leading to high recurrence rates of cancer.^900^ Therefore, combination of anti-inflammatory strategies with cancer treatment have improved the anti-cancer effect in some clinical cases and in vivo experiments, such as COX-2 inhibitors,^901^ NSAIDs,^902–904^ LOX Inhibitors,^905^ and statins.^906^ Besides, precision medicine, also be called personalized medicine, should also be taken into account in different inflammatory responses of cancer patients during the anti-tumor process and adopt personalized therapeutic strategies targeting inflammation.

As we underlined the several mechanisms of the interaction between inflammation and cancer, the essence of inflammation-targeting cancer therapy is to promote cancer-inhibiting inflammation and inhibit cancer-promoting inflammation, while the biggest difficulty of treatment is to maintain the balance of inflammation. Except for the targets mentioned above, there are numerous molecules involved in the regulation of inflammation and cancer, like intestinal microbiota and their metabolites. Besides, anti-tumor therapies targeting inflammation should be incorporated into precision therapy. However, applying these theories to clinical cancer therapy is still a long way off. In addition, a large number of studies would continue to contribute to the reinforcement of the theoretical basis of inflammation-targeting cancer treatment, constantly updating the field.
